# Supplementary material for: Multiple orthoflaviviruses secrete sfRNA in mosquito saliva to promote transmission by inhibiting MDA5-mediated early interferon response
Source: Nat Commun. 2026 Aug 29;17:10323. doi: 10.1038/s41467-026-77288-4 (PMC13623828; doi:10.1038/s41467-026-77288-4)
Supplement: Supplementary file 2 [file 41467_2026_77288_MOESM2_ESM.pdf]

nature portfolio

Corresponding author(s): Julien Pompon

Last updated by author(s): Aug 5, 2026

Reporting Summary

Nature Portfolio wishes to improve the reproducibility of the work that we publish. This form provides structure for consistency and transparency in reporting. For further information on Nature Portfolio policies, see our Editorial Policies and the Editorial Policy Checklist.

Statistics

For all statistical analyses, confirm that the following items are present in the figure legend, table legend, main text, or Methods section.

n/a	Confirmed	
☐	☒	The exact sample size (*n*) for each experimental group/condition, given as a discrete number and unit of measurement
☐	☒	A statement on whether measurements were taken from distinct samples or whether the same sample was measured repeatedly
☐	☒	The statistical test(s) used AND whether they are one- or two-sided<br>*Only common tests should be described solely by name; describe more complex techniques in the Methods section.*
☐	☒	A description of all covariates tested
☐	☒	A description of any assumptions or corrections, such as tests of normality and adjustment for multiple comparisons
☐	☒	A full description of the statistical parameters including central tendency (e.g. means) or other basic estimates (e.g. regression coefficient) AND variation (e.g. standard deviation) or associated estimates of uncertainty (e.g. confidence intervals)
☐	☒	For null hypothesis testing, the test statistic (e.g. *F*, *t*, *r*) with confidence intervals, effect sizes, degrees of freedom and *P* value noted<br>*Give P values as exact values whenever suitable.*
☒	☐	For Bayesian analysis, information on the choice of priors and Markov chain Monte Carlo settings
☒	☐	For hierarchical and complex designs, identification of the appropriate level for tests and full reporting of outcomes
☒	☐	Estimates of effect sizes (e.g. Cohen's *d*, Pearson's *r*), indicating how they were calculated

Our web collection on statistics for biologists contains articles on many of the points above.

Software and code

Policy information about availability of computer code

Data collection	n/a
Data analysis	n/a

For manuscripts utilizing custom algorithms or software that are central to the research but not yet described in published literature, software must be made available to editors and reviewers. We strongly encourage code deposition in a community repository (e.g. GitHub). See the Nature Portfolio guidelines for submitting code & software for further information.

Data

Policy information about availability of data

All manuscripts must include a data availability statement. This statement should provide the following information, where applicable:

- Accession codes, unique identifiers, or web links for publicly available datasets
- A description of any restrictions on data availability
- For clinical datasets or third party data, please ensure that the statement adheres to our policy

All data supporting the findings of this study are available within the paper and its Supplementary Information. Source Data are provided with the paper.

Research involving human participants, their data, or biological material

Policy information about studies with human participants or human data. See also policy information about sex, gender (identity/presentation), and sexual orientation and race, ethnicity and racism.

Reporting on sex and gender	Skin explant were collected from a female.
Reporting on race, ethnicity, or other socially relevant groupings	Ethnicity was caucasian.
Population characteristics	age = 45 year-old; BMI = 25; photype: III
Recruitment	Recruitment was voluntary and written consent was requested.
Ethics oversight	Ethical approval for the use of human skin explant was provided by the French ministry of higher education and research to Biopredic (permission number: AV-2023-5431). Informed consent was requested.

Note that full information on the approval of the study protocol must also be provided in the manuscript.

Field-specific reporting

Please select the one below that is the best fit for your research. If you are not sure, read the appropriate sections before making your selection.

☒ Life sciences ☐ Behavioural & social sciences ☐ Ecological, evolutionary & environmental sciences

For a reference copy of the document with all sections, see nature.com/documents/nr-reporting-summary-flat.pdf

Life sciences study design

All studies must disclose on these points even when the disclosure is negative.

Sample size	For mosquito samples, sample size was selected to obtain a significant differences between saliva and salivary glands.<br>For cell experiments, a minimum sample size of 6 was selected and included 3 wells obtained from two cell batches.<br>For mouse experiment, sample size was selected to obtain significant differences in survival.
Data exclusions	No data was excluded.
Replication	Experiments performed in cells were repeated at least 6 times, by combining three well repeats from two cell batch experiments. Experiments with mosquitoes were conducted with about 20 mosquito repeats, same for saliva collection. Mouse experiments were conducted with 14 mice per condiction housed in different cages.
Randomization	Allocation of samples was randomized.
Blinding	Symptom evaluation for mice was done after blinding the conditions. Unblinding was achieved once all results were analyzed. Analyses of gRNA and sfRNA quantification was done using a thermocycler, which cannot be susceptible to biases. Therefore, quantification of gRNA and sfRNA was not blinded.

Reporting for specific materials, systems and methods

We require information from authors about some types of materials, experimental systems and methods used in many studies. Here, indicate whether each material, system or method listed is relevant to your study. If you are not sure if a list item applies to your research, read the appropriate section before selecting a response.

Materials & experimental systems

n/a	Involved in the study	
☐	☒	Antibodies
☐	☒	Eukaryotic cell lines
☒	☐	Palaeontology and archaeology
☐	☒	Animals and other organisms
☒	☐	Clinical data
☒	☐	Dual use research of concern
☒	☐	Plants

Methods

n/a	Involved in the study	
☒	☐	ChIP-seq
☒	☐	Flow cytometry
☒	☐	MRI-based neuroimaging

Antibodies

Antibodies used	1:4,000 anti-MDA5 antibody (21775-1-AP, Proteintech); 1:400 anti-Actin (MA5-11869, Invitrogen); anti-rabbit IgG HRP-linked antibody (Cell signaling).
Validation	siRNA mediated silencing was performed to validate the antibody.

Eukaryotic cell lines

Policy information about cell lines and Sex and Gender in Research

Cell line source(s)	Aedes albopictus C6/36 and Human hepatocyte Huh7 cells were obtained from Cell Lines Services GmbH and Huh7.5 cells were kindly provided by Paul D. Biensiasz (The Rockefeller University, New York, NY, USA). Vero (CCL-81), HFF1 (SCRC-1041) and U937 (CRL-1593.2) cells were obtained from ATCC.
Authentication	Authentication was done by the commercial provider by PCR.
Mycoplasma contamination	All cells were tested negative for mycoplasma contamination using PCR method.
Commonly misidentified lines (See ICLAC register)	None of the cells were part of the commonly misidentified lines.

Animals and other research organisms

Policy information about studies involving animals; ARRIVE guidelines recommended for reporting animal research, and Sex and Gender in Research

Laboratory animals	5-7 week-old C57BL/6J male mice (Mus musculus) were purchased from Charles-River (France).<br>Experiments with Culex mosquitoes were carried out using the Culex quinquefasciatus SLAB strain, collected in California, USA, and obtained from the Institut des Sciences de l'Evolution in Montpellier, France. Experiments with Aedes mosquitoes were carried out using the Aedes aegypti Bora-Bora strain collected in French Polynesia in 1980.
Wild animals	n/a
Reporting on sex	Only male mice were used. We reasoned that showing an effect in one sex was sufficient to demonstrate our hypothesis.
Field-collected samples	n/a
Ethics oversight	Animal protocols were approved by the national ethical committee from the ministry of higher education and research in France (permission numbers: 43466; 31273) and IRD ethical committee.

Note that full information on the approval of the study protocol must also be provided in the manuscript.

Plants

Seed stocks	n/a
Novel plant genotypes	n/a
Authentication	n/a
