# Supplementary material for: Multiple orthoflaviviruses secrete sfRNA in mosquito saliva to promote transmission by inhibiting MDA5-mediated early interferon response
Source: Nat Commun. 2026 Aug 29;17:10323. doi: 10.1038/s41467-026-77288-4 (PMC13623828; doi:10.1038/s41467-026-77288-4)
Supplement: Supplementary file 3 [file 41467_2026_77288_MOESM3_ESM.pdf]

nature portfolio

Peer Review File

Multiple orthoflaviviruses secrete sfRNA in mosquito saliva to promote transmission by inhibiting MDA5-mediated early interferon response

Corresponding Author: Dr Julien Pompon

This file contains all reviewer reports in order by version, followed by all author rebuttals in order by version.

Version 2:

Reviewer comments:

Reviewer #1

(Remarks to the Author)
The authors previously discovered that that dengue virus produces a non-coding subgenomic orthoflaviviral RNA (sfRNA) with immune-suppressing properties inside lipid vesicles released in mosquito saliva. However, they did not show how this affects virus transmission or the mechanism behind it. In this new study, the authors investigate the secretion of sfRNA-containing lipid vesicles from two other orthoflaviviruses and their role in enhancing virus infection in various human skin cells, as well as suppressing the interferon (IFN) response. The research involved infecting Culex mosquitoes with West Nile virus and Aedes mosquitoes with Zika virus. Analyses of their salivary glands and saliva samples confirmed the presence of both sfRNA and genomic RNA gRNA. Treatment with MNase and Triton X-100 degraded these RNAs, suggesting that they are contained within lipid vesicles. Critically, the injection of sfRNA into human cell lines and in vivo mouse models enhanced viral infection, leading to worsened disease outcomes. The study also found that transfection with sfRNA suppresses the initial IFN response by disrupting MDA5-mediated signaling pathways. This study potentially offers important novel insights into the function of sfRNA as a conserved transmission-enhancing factor across orthoflaviviruses. Nevertheless, several key issues require clarification to fully establish the relevance of these conclusions.

Major points
A. In their experiments, the authors observed no correlation between sfRNA and gRNA levels in saliva from WNV-infected Culex and ZIKV-infected Aedes following Triton X-100 and MNase treatments (Figure 2e and i), leading them to conclude that gRNA and sfRNA are packaged in separate salivary lipid vesicles. However, the limited sample size for the saliva analysis and the narrow variability on the y-axis for WNV and on the x-axis for ZIKV may affect the statistical robustness of this conclusion. Additionally, differences in MNase sensitivity between sfRNA and gRNA might also contribute to the observed lack of correlation. Therefore, the claim that gRNA and sfRNA are packaged in distinct salivary lipid vesicles should be reconsidered until further supporting evidence is obtained.
B. In Figures 3 and 4, the authors examined the role of saliva sfRNA in virus transmission and its impact on the IFN response. It is important to note that both saliva sfRNA and infectious virus particles can influence virus transmission to human skin cells and their IFN responses. Therefore, controlling for the infectious virus inoculum in these experiments is essential. Figure S10 shows the correlation between the sfRNA:gRNA ratio and the gRNA:PFU ratio, but with only four data points and limited variability on the y-axis, it remains unclear whether gRNA reliably indicates saliva infectiousness. It would be more informative if Figure S10 showed PFU as a function of gRNA across varying sfRNA levels. Additionally, the methods section does not describe the plaque assay and does not specify how the gRNA:PFU ratio is calculated, leaving open the question of whether the PFU measurement fully accounts for the influence of sfRNA. Clarifying the methods used to measure saliva infectiousness is needed to interpret the findings. Finally, normalizing sfRNA levels by saliva infectiousness, rather than by gRNA (as in Figures 3d and 3f), would provide a clearer interpretation of the data.

Minor points
1. Lines 68-69: Not all orthoflavivirus transmission events are through a mosquito bite (e.g., ZIKV can be transmitted sexually and vertically), so please modify this sentence accordingly.
2. Line 94 : This sentence should be revised to ensure clarity regarding abbreviations: "West Nile virus (WNV) and Zika virus (ZIKV)".
3. Line 100 : The WHO declared Zika virus a Public Health Emergency of International Concern on February 1, 2016.

Please correct the date.
4. Figure 1 : Statistical test are missing in most of the panels, they should be included.
5. Figure 1b and 1c: Please provide an explanation for why the detection rate of sfRNA is higher than that of gRNA in both the SG and saliva samples. If sfRNA is generated in the SG, one would expect to find more gRNA than sfRNA.
6. Figure 1d: To prevent any potential misunderstanding, please include samples with an sfRNA:gRNA ratio equal to zero in the graphs, similar to how they are presented in Figure 3a.
7. Lines 140-141: It would be helpful to provide additional evidence demonstrating the generation of sfRNA in the SG. For instance, is it feasible to detect sfRNA and gRNA at different time points to support this observation?
8. Similar to Figure 1a, it would be useful to present the patterns of ZIKV sfRNAs using Northern blot analysis.
9. Showing the correlation between sfRNA and gRNA for ZIKV, as was done for WNV (Figure 1e and 1f), would offer further evidence for the generation of sfRNA in the SG and different release pathway.
10. Line 160: This conclusion is not supported by the data and should be revised. Thoracic inoculation bypasses but does not necessarily prevent midgut infection.
11. Figure 2: As suggested above in major point A, it may be challenging to definitively conclude that sfRNA and gRNA exist in separate particles due to potential differences in MNase sensitivity. The authors should consider revising the results section to reflect this limitation.
12. Figure 3b and 3c: Please provide insight into why there is no observed increase in the measurements from 24 to 48 hours post infection.
13. Figure 3d and f: As suggested above in major point B, if gRNA is not indicative of saliva infectiousness, normalization of the sfRNA levels based on saliva infectiousness could provide a clearer analysis.
14. Figure 4a-d: Similar to Figure 3d and 3f, it would be more relevant to normalize sfRNA levels by saliva infectiousness.
15. To improve readability, it would be beneficial to standardize the labeling of the y-axis (e.g., using log10, 10x, or fold change) as well as the line scales consistently throughout the manuscript.

Reviewer #2

(Remarks to the Author)
I co-reviewed this manuscript with one of the reviewers who provided the listed reports. This is part of the Nature Communications initiative to facilitate training in peer review and to provide appropriate recognition for Early Career Researchers who co-review manuscripts.

Reviewer #3

(Remarks to the Author)
The article describes the role that sfRNA produced from the flavivirus 3'UTR contained in extracellular vesicles in mosquito saliva, plays in enhancing viral infection. The study reports the identification of sfRNA molecules being contained in EVs similar to a previous study by the authors. Authors employed various techniques to demonstrate and quantity sfRNA produced in culex and aedes mosquitoes saliva EVs for WNV and ZIKV respectively. They proceeded to show that increasing amounts of sfRNA-EVs in saliva increase early replication of the virus in both cell and skin models. They further added by showing that co-injection of WNV and sfRNA in mice increased disease severity and mortality compared to co-injection of WNV and control RNA. They then went on to show that addition of sfRNA had mixed results in inhibiting IFN response in different cell lines but was effective in the skin explants. Finally, authors concluded by showing that sfRNA could inhibit MDA5 signalling pathways as the mechanisms for inhibition.
The manuscript has a number of major deficiencies in methodologies (as listed below) leading to questioning of the main conclusions drawn from the results.

Major Concerns
1. There is no conclusive evidence of sfRNAs in mosquito saliva provided. Authors only show northern blot for sfRNA using total RNA extracted from whole mosquitoes. Authors should also provide northern blot of RNA from saliva with detectable sfRNA. Pulled saliva samples can be used to ensure sufficient amount sfRNA for northern blot detection.
2. Quantification of sfRNA by comparative RT-qPCR with genomic viral RNA has significant flaws in its method. Authors have stated in their previous paper that this may overestimate the sfRNA concentrations. It does not allow for differences in processing of genomic RNA to sfRNA or efficiency in RT and PCR. Direct quantification methods which quantify sfRNA without a need to quantify viral genomic RNA should be established similar to those found in studies involving small non-coding RNA. This may involve ligating adapter to 5' end of sfRNA following by RT-PCR with primers complementary of 3' end of sfRNA and to the adapter-5'end of sfRNA junction.
3. Figure 4A uses saliva extracted from mosquitos where differences in viral inoculation may occur (as stated by authors). Therefore, quantities of infectious virus (i.e. viral titres) should also be included for analysis and to be used for normalisation.
4. In Fig 4e-t and Fig 5 b-d authors used extremely low MOI (MOI=0.005) of infection with sfRNA-producing virus and transfected extremely high copies of sfRNA (10e10 copies) resulting in inconclusive results because very few cells would be infected and mount IFN response. Rescue of IFN response by sfRNA using higher MOI infection with sfRNA deficient viruses would provide more appropriate analysis because this will allow to standardise cell response to the virus in the absence of sfRNA and adequately access the role of exogenously added sfRNA on these responses. Also authors need to provide an evidence that sfRNA is actually being transfected and present in transfected cells, e.g. transfect FAM-labelled sfRNA.
5. Previous studies have implicated STAT1 inhibition by sfRNA as an inhibitor of IFN pathways. Why did authors not investigate this mechanism as well?

Version 3:

Reviewer comments:

Reviewer #1

(Remarks to the Author)
The authors have addressed all previously raised concerns in a convincing manner. Their thorough revision has substantially improved the quality of the manuscript.

Reviewer #2

(Remarks to the Author)
I co-reviewed this manuscript with one of the reviewers who provided the listed reports. This is part of the Nature Communications initiative to facilitate training in peer review and to provide appropriate recognition for Early Career Researchers who co-review manuscripts.

Reviewer #3

(Remarks to the Author)
Major concerns 1 and 2 – evidence for sfRNA in saliva and quantification of sfRNA.
These are still the major issues with this manuscript.

Authors provided very poor-quality Northern blot presumed to show sfRNA in a pooled saliva sample from 100 mosquitoes. There is no distinct band in the blot that would convincingly demonstrate presence of sfRNA, which authors themselves admit in their response.

The authors then went to test several other methods for sfRNA detection with most of them failing. However, one of the methods, based on splinted-ligation RT-PCR appeared to work (new panel J in the figure). The addition of panel J goes some way to alleviating concerns about detecting sfRNA directly, however authors have not addressed the concerns over quantification. There are limited details about the protocol, including amount of input RNA used in the reverse transcription, as well as the number of cycles used in the PCR. Additionally, the amplification in the sample where key enzyme, ligase, was absent raises concerns about non-specific amplification. An additional control using synthetic RNA with the 5' end further upstream (as to simulate genomic RNA) would also improve the validity of the experiment. Were the products sequenced to confirm generation of the appropriate fragments?

Although the authors have added this figure, they have not used this method to quantify the virus and still rely of the subtractive PCR methods in the original manuscript. Why did authors not use this direct quantification method to quantify sfRNA in their saliva pools? Variation in band intensity of direct sfRNA RT-PCR in the new panel J compared to the original estimates (Line 191) suggests an error in one of these techniques.

Major concern 4 – use of low MOI and detection of transfected sfRNA

MOI - the suggested to be used higher MOI emulates similar results. However, It doesn't appear that virus gRNA changes much in the higher MOI infections, did the authors do virus quantification?

sfRNA transfection control - The RT-PCR of the transfected control RNA and sfRNAs is adequate to support this but a negative control would add greater clarity. Authors could test cells transfected with control RNA using sfRNA-specific primers and vice versa as negative controls.

Version 4:

Reviewer comments:

Reviewer #3

(Remarks to the Author)
Authors provided adequate response and data for quantification of sfRNA, however the Northen blot detection of sfRNAs and our request for negative controls in sfRNA-transfected cells were not adequately addressed.

Northern bolts.
Bands labelled as sfRNA1 in saliva samples from WNV-infected mosquitoes in Fig S6 and in whole mosquito sample from ZIKV-infected mosquitoes in Figs S8 are still not convincing at all. These should be ether redone with different detection methods (as for example in Pallares et al 2020 and Slonchak et al 2020) to convincingly show detection of sfRNAs by Northern blots or removed from the paper with claims on detection of sfRNA by Northern blot also removed from the text.

Negative sfRNA transfection controls.
While authors performed additional experiment to include amplification of in vitro transcribed sfRNA with control RNA

primers and in vitro transcribed control RNA with sfRNA primers, no data on requested transfection controls for the uptake of sfRNA and detection with control primers as well uptake of control RNA and detection with sfRNA primers were presented. This was requested to adequately demonstrate consistent uptake of the RNAs by the cells and their specific detection in transfected cell environment. This would increase confidence in the specificity of sfRNA detection in transfected cells by RT-PCR.

Open Access This Peer Review File is licensed under a Creative Commons Attribution 4.0 International License, which permits use, sharing, adaptation, distribution and reproduction in any medium or format, as long as you give appropriate credit to the original author(s) and the source, provide a link to the Creative Commons license, and indicate if changes were made.

In cases where reviewers are anonymous, credit should be given to 'Anonymous Referee' and the source.

The images or other third party material in this Peer Review File are included in the article's Creative Commons license, unless indicated otherwise in a credit line to the material. If material is not included in the article's Creative Commons license and your intended use is not permitted by statutory regulation or exceeds the permitted use, you will need to obtain permission directly from the copyright holder.

To view a copy of this license, visit https://creativecommons.org/licenses/by/4.0/

Answers to Reviewers comments.

We sincerely thank the reviewers for their time and comments, which have improved the quality and strength of the manuscript. We have carefully addressed all comments raised in the detailed point-by-point responses provided below in blue.

Julien Pompon

In particular, please provide **stronger evidence for the presence of sfRNA in mosquito saliva (only) by including Northern Blot data** as suggested by reviewer #3.

We generated a Northern blot to detect sfRNA in saliva from WNV-infected mosquitoes by pooling saliva from several hundred individuals. However, given the limited sensitivity of NB, the sfRNA band was extremely faint, and we consider that these data alone do not provide sufficiently robust evidence. To more convincingly support the presence of sfRNA in saliva, we therefore developed a sensitive molecular assay based on splinted-ligation RT–PCR (SL-RT-PCR), a strategy commonly used to detect mRNA decay intermediates. Using SL-RT-PCR validated with synthetic sfRNA, we successfully detected sfRNA in multiple independent saliva pools, providing strong evidence for its presence in mosquito saliva.

Additionally, we performed a Northern blot analysis of sfRNA in ZIKV-infected mosquitoes, showing that sfRNA1 represents the predominant species in mosquitoes.

Further, you will need to provide more **solid data for sfRNA quantification** (reviewer #3) and clarify how the **gRNA:sfRNA ratios are calculated (reviewer #1).**

Leveraging strategies used to detect mRNA decay products, we have developed a novel direct sfRNA detection tool, which we validated with synthetic sfRNA. With this molecular technology, we directly detected sfRNA in saliva from WNV-infected mosquitoes. By breaking a long-standing technological limit in sensitive sfRNA detection, we are confident that this technical development will be of great benefit to the community and to strengthen our main message that sfRNA is present in saliva.

Additionally, we clarified our methods for sfRNA quantification using the initial subtractive RT-qPCR method.

Eventually, you will need to improve your transmission experiments by **including and controlling for viral titers** as suggested by all referees and **improve your mechanistic data along the lines** suggested by reviewer #3.

In the initial version, viral gRNA levels were used to normalize infection levels across saliva samples. Molecular detection of gRNA requires minimal sample volume, whereas cell-based viral titration requires substantially larger volumes, precluding the use of the same saliva pool both for infection quantification and as the inoculum for functional assays. To control for viral titers, we have now established a strong correlation between gRNA levels and infectious particle counts using 9 independent pooled saliva samples.

Additionally, we reinforced the mechanistic support for our findings by reproducing the functional impact of sfRNA transfection on infection levels at a 100-fold higher MOI, as suggested the reviewers. Importantly, we also quantified innate immune activation in this condition and obtained a comparable inhibition of the IFN response to that observed at the initial lower MOI.

Below, line numbers as indicated for changes are in the version with accepted changes.

REVIEWER COMMENTS

Reviewer #1 (Remarks to the Author):

The authors previously discovered that that dengue virus produces a non-coding subgenomic orthoflaviviral RNA (sfRNA) with immune-suppressing properties inside lipid vesicles released in mosquito saliva. However, they did not show how this affects virus transmission or the mechanism behind it. In this new study, the authors investigate the secretion of sfRNA-containing lipid vesicles from two other orthoflaviviruses and their role in enhancing virus infection in various human skin cells, as well as suppressing the interferon (IFN) response. The research involved infecting Culex mosquitoes with West Nile virus and Aedes mosquitoes with Zika virus. Analyses of their salivary glands and saliva samples confirmed the presence of both sfRNA and genomic RNA gRNA. Treatment with MNase and Triton X-100 degraded these RNAs, suggesting that they are contained within lipid vesicles. Critically, the injection of sfRNA into human cell lines and in vivo mouse models enhanced viral infection, leading to worsened disease outcomes. The study also found that transfection with sfRNA suppresses the initial IFN response by disrupting MDA5-mediated signaling pathways. This study potentially offers important novel insights into the function of sfRNA as a conserved transmission-enhancing factor across orthoflaviviruses. Nevertheless, several key issues require clarification to fully establish the relevance of these conclusions.

We are extremely thankful to the reviewer for acknowledging the important novel insights we provide on salivary sfRNA. We have addressed all the points raised by generating multiple new datasets.

Major points

A. In their experiments, the authors observed no correlation between sfRNA and gRNA levels in saliva from WNV-infected Culex and ZIKV-infected Aedes following Triton X-100 and MNase treatments (Figure 2e and i), leading them to conclude that gRNA and sfRNA are packaged in separate salivary lipid vesicles. However, the limited sample size for the saliva analysis and the narrow variability on the y-axis for WNV and on the x-axis for ZIKV may affect the statistical robustness of this conclusion.

Experiments with saliva are notoriously difficult, even more so when this includes multiple-step experiments for RNAse resistance. To answer reviewer's comment, we have conducted three more repeats of RNAse resistance assay with both WNV-infected *Culex* saliva and ZIKV-infected *Aedes* saliva. The new figures 2e and 2i confirm that there is no correlation between sfRNA and gRNA quantities after triton/RNase treatment. Of note, ZIKV samples had two conditions without detectable gRNA and

detected sfRNA, which are not included in the fig. 2i. These added results strengthen our hypothesis that sfRNA and gRNA are in different lipid-based vesicles.

e

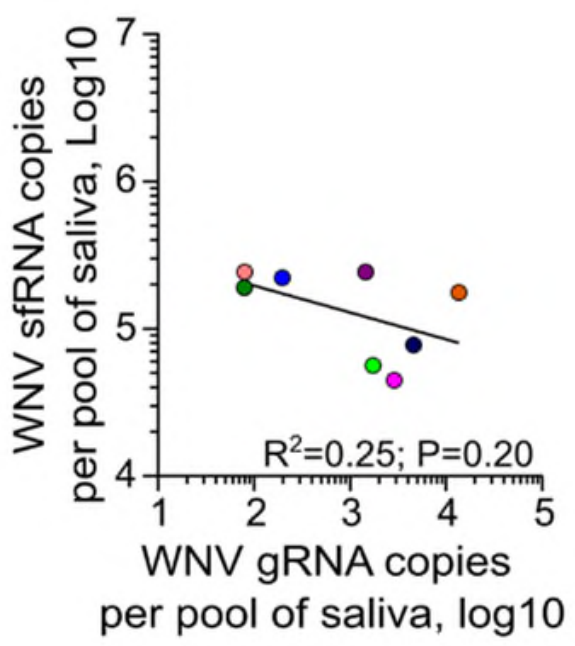

i

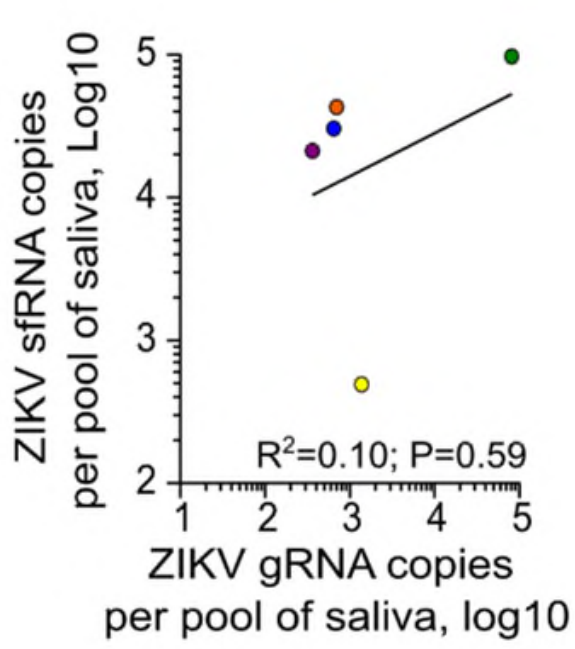

Additionally, differences in MNase sensitivity between sfRNA and gRNA might also contribute to the observed lack of correlation. Therefore, the claim that gRNA and sfRNA are packaged in distinct salivary lipid vesicles should be reconsidered until further supporting evidence is obtained.

We agree that differences in MNase and RNase resistance between sfRNA and gRNA could bias our interpretation. To address this comment, we evaluated the resistance of a viral RNA control (initially used as a control for sfRNA injection) to both MNase and RNase. The assay was conducted in the presence of uninfected mosquito saliva, either from *Culex* for MNase or from *Aedes* for RNase, for coherence with sfRNA RNAse resistance assay. In the new S12a,b Fig., the sensitivity of the viral control RNA is clearly demonstrated with nearly all of the 10^7 input copies of Control RNA being degraded. This observation has now been incorporated in the results section:

(l. 224-27): "We showed that micrococcal nuclease (MNase) degraded in vitro transcribed WNV sfRNA (Fig. 2b) (RNase A/T1 was inefficient in degrading WNV sfRNA) and a viral RNA control fragment (S12a Fig.)"

And l. 240-241: "After confirming the degradation of in vitro-transcribed ZIKV sfRNA (Fig. 2f) and viral control RNA (S12b Fig.)"

And in the method section (l. 649).

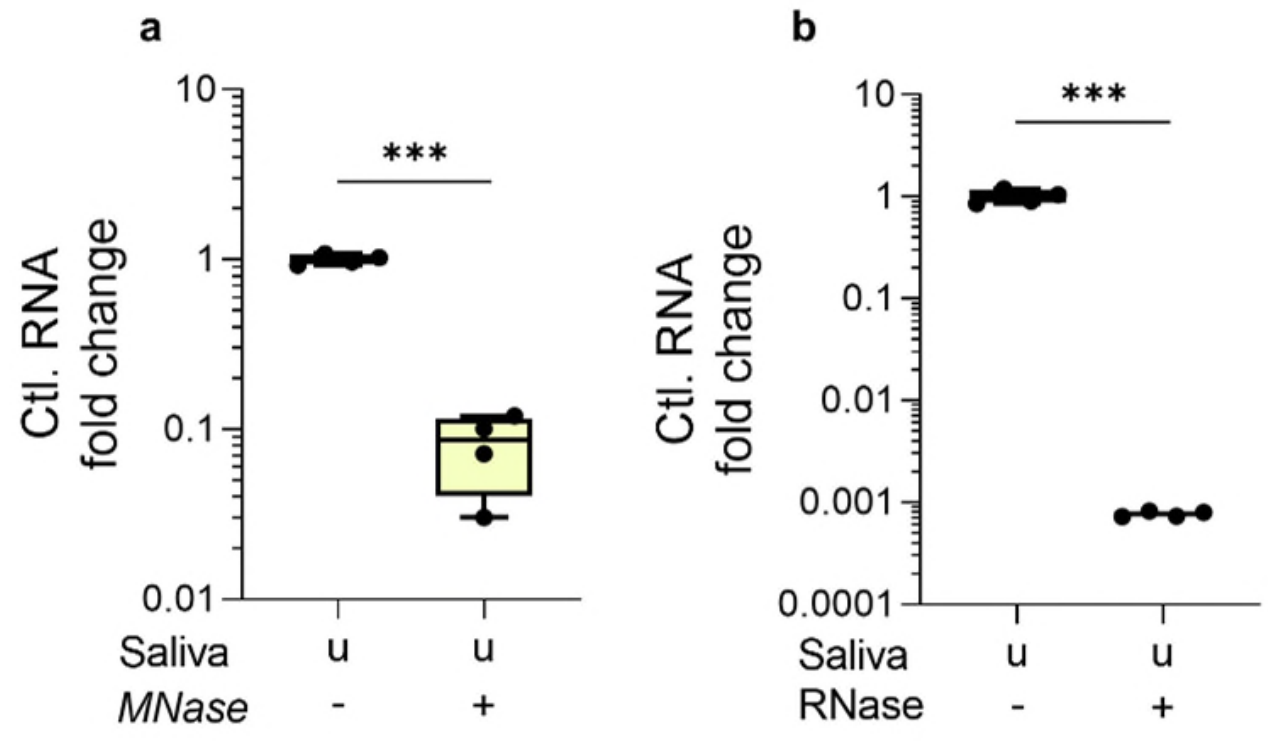

S11 Fig. (Related to Fig. 2) | a-b MNase and RNase degrade a viral RNA control. Boxplots indicate median ± lower and higher quartiles. N, 4. ***, p < 0.001 according to T-test.

Nonetheless, we acknowledge that this only represents a proxy for complete genomic RNA degradation and modulated our conclusion which now reads:

l. 253-255: "Altogether, the analysis of saliva from two different mosquito genera infected with different orthoflaviviruses **suggest** the conservation of sfRNA secretion within salivary lipid vesicles across multiple orthoflaviviruses. "

B. In Figures 3 and 4, the authors examined the role of saliva sfRNA in virus transmission and its impact on the IFN response. It is important to note that both saliva sfRNA and infectious virus particles can influence virus transmission to human skin cells and their IFN responses. Therefore, controlling for the infectious virus inoculum in these experiments is essential. Figure S10 shows the correlation between the sfRNA:gRNA ratio and the gRNA:PFU ratio, but with only four data points and limited variability on the y-axis, it remains unclear whether gRNA reliably indicates saliva infectiousness. It would be more informative if Figure S10 showed PFU as a function of gRNA across varying sfRNA levels.

We agree that controlling for the inoculum dose is essential. In the initial version, we therefore used molecular quantification of viral genomic RNA (gRNA) to normalize gRNA copy numbers across the different salivary inocula. Infection levels were quantified by RT–qPCR rather than by cell-based titration, as the molecular approach requires minimal sample volume and allowed the same sample to be used both for infection quantification and as the inoculum – a requirement for our experimental approach.

To address the reviewer's comment, we now provide the correlation between gRNA levels and infectious particles quantified by a cell-based titration assay (PFU) in Fig. S15a (see below), including the initial four samples and five additional saliva samples. These results show a strong positive correlation between gRNA

levels and PFU in saliva from WNV-infected mosquitoes, supporting the use of gRNA as a reliable proxy for infection level.

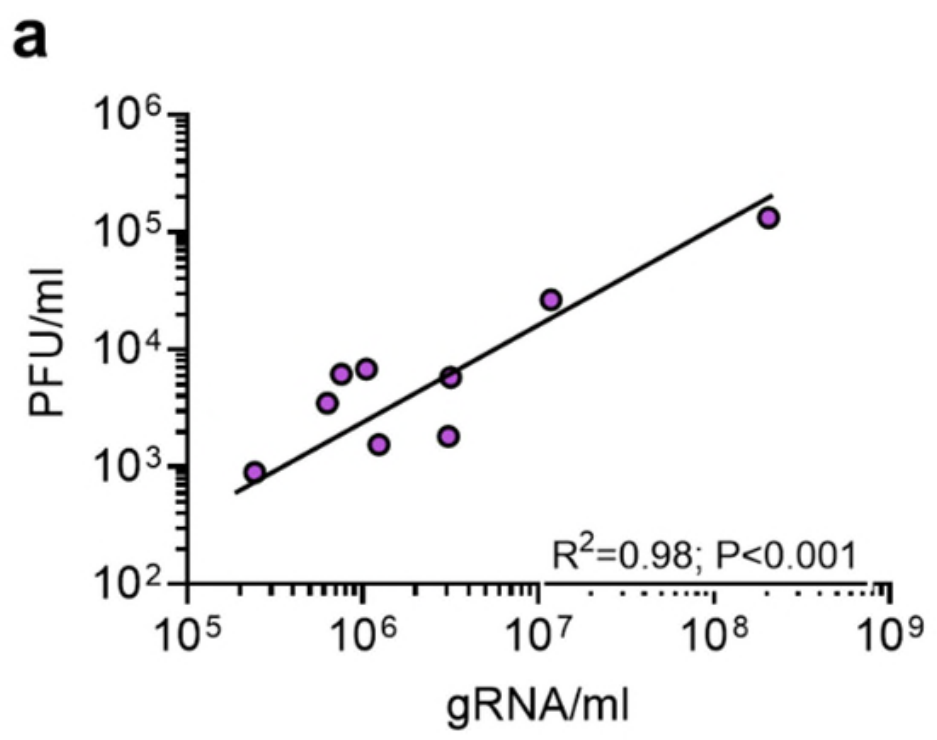

We added this new information in the results section (l. 265-269):

"To support the normalization of saliva inoculum dose based on gRNA quantities, we showed that gRNA quantities were proportional to saliva infectiousness as measured by particle forming unit (PFU) ($R^2 = 0.98$; $p < 0.001$; S15a Fig.) »

Additionally, the methods section does not describe the plaque assay and does not specify how the gRNA:PFU ratio is calculated, leaving open the question of whether the PFU measurement fully accounts for the influence of sfRNA. Clarifying the methods used to measure saliva infectiousness is needed to interpret the findings.

We thank the reviewer for pointing out this oversight. We have now added the protocol for virus titration (l. 663-670) followed by the calculation for gRNA:PFU ratio (l. 671): "gRNA:PFU ratio was calculated by dividing the gRNA copy number quantified as detailed above to the PFU number within the same saliva."

Finally, normalizing sfRNA levels by saliva infectiousness, rather than by gRNA (as in Figures 3d and 3f), would provide a clearer interpretation of the data.

We acknowledge the reviewer's point and now provide a more robust justification for normalizing saliva infectiousness in terms of gRNA copies. In the new S15 Fig., we demonstrate that gRNA levels are strongly correlated with saliva infectiousness, as measured by PFU ($R^2 = 0.98$; $p < 0.001$). To further strengthen this correlation, we quantified new saliva samples, increasing the total number to 9 saliva repeats. In Figures 3d and 3f, we chose to normalize saliva infectiousness using gRNA levels because gRNA quantification requires significantly less starting saliva material than plaque assays. This approach therefore preserved sufficient volumes of precious infectious saliva for subsequent infection experiments.

Minor points

1. Lines 68-69: Not all orthoflavivirus transmission events are through a mosquito bite (e.g., ZIKV can be transmitted sexually and vertically), so please modify this sentence accordingly.

Agreed and we now added: "although alternative transmission routes such as sexual and maternal transmissions are possible "(l. 72)

2. Line 94 : This sentence should be revised to ensure clarity regarding abbreviations: "West Nile virus (WNV) and Zika virus (ZIKV)".

Modified as suggested.

3. Line 100 : The WHO declared Zika virus a Public Health Emergency of International Concern on February 1, 2016. Please correct the date.

Corrected. Sorry for the error.

4. Figure 1 : Statistical test are missing in most of the panels, they should be included.

Statistical tests were included in Figure 1 whenever significant. Statistical significance was added for gRNA and sfRNA difference between SG and saliva.

5. Figure 1b and 1c: Please provide an explanation for why the detection rate of sfRNA is higher than that of gRNA in both the SG and saliva samples. If sfRNA is generated in the SG, one would expect to find more gRNA than sfRNA.

Indeed, since sfRNA is derived from gRNA in SG, the number of sfRNA-positive samples should not exceed that of gRNA-positive samples. The apparent discrepancy highlighted in the figure arises from the method used to calculate the sfRNA detection rate. As detailed in method section: "sfRNA detection rate was calculated as the number of samples with detectable amount of sfRNA only among gRNA-positive samples." (l. 622-623). With this approach, the sfRNA detection rate exclusively reflects sfRNA detection within gRNA-positive samples.

To prevent misunderstanding, we now added in the Figure 1 legend: "sfRNA detection rate was calculated among **gRNA-positive** samples." and mentioned in the results section that "we detected sfRNA in 93% of **gRNA-positive** SG [...] and in 100% of **gRNA-positive** saliva" (l. 143).

6. Figure 1d: To prevent any potential misunderstanding, please include samples with an sfRNA:gRNA ratio equal to zero in the graphs, similar to how they are presented in Figure 3a.

We thank the reviewer for the suggestion. However, salivary sfRNA concentrations exhibit significant variability between experiments, which we do not understand and do not know how to control. In our initial experiments with oral infection for Figure 1a-f, we did not observe any saliva samples with undetectable sfRNA levels. In contrast, when repeating oral infection for collecting saliva for infection, there were saliva samples with undetectable sfRNA. This difference between the two sets of samples (Fig 1 vs Fig 3) explains the lack of samples with no detectable sfRNA in Fig 1. All samples collected for Figure 1 were included.

7. Lines 140-141: It would be helpful to provide additional evidence demonstrating the generation of sfRNA in the SG. For instance, is it feasible to detect sfRNA and gRNA at different time points to support this observation?

While determining whether sfRNA is generated in the salivary glands is an intriguing question, addressing this question is highly challenging. We understand that the reviewer suggests performing a kinetic quantification of sfRNA and gRNA to support a correlated production of the two RNA molecules. However, we do not believe this approach would effectively answer the question for several reasons. First, as mentioned in the Introduction, sfRNA is derived from gRNA degradation by host exoribonuclease Xrn1[1], a highly processive enzyme that cleaves 38–55 phosphodiester bonds per second[2]. As a result, gRNA and sfRNA are generated almost simultaneously. Second, no established link exists between gRNA and sfRNA levels. This means that an increase in gRNA does not necessarily lead to higher sfRNA levels. In contrast, some evidence even suggests that slower gRNA production may actually favor sfRNA biogenesis[3].

We regret that we cannot be more supportive of this request. However, we believe that this does not undermine the main conclusions of the study, which focus on the role of salivary sfRNA in transmission.

8. Similar to Figure 1a, it would be useful to present the patterns of ZIKV sfRNAs using Northern blot analysis.

To answer the reviewer's request, we developed NB against ZIKV sfRNA in our lab and applied it on ZIKV-infected mosquitoes. Although the sensitivity was low, we could still detect a single band at the expected size for ZIKV sfRNA, as in previous paper [4]. The new results are presented in Fig. S7 (see below) and detailed in the results section:
(l. 200-202): "we inoculated Aedes mosquitoes with ZIKV and collected SG and saliva at 7 dpi. In whole mosquitoes, NB showed the presence of a single sfRNA band at the expected size (S7 Fig.), as previously detected in *Ae. aegypti* [45].".

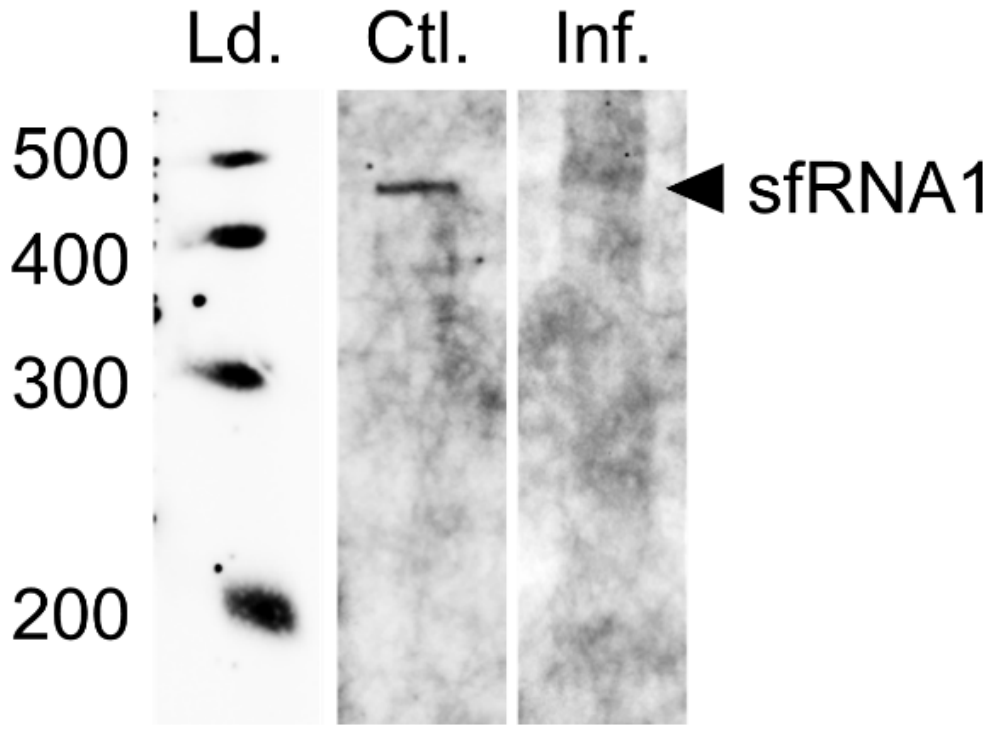

9. Showing the correlation between sfRNA and gRNA for ZIKV, as was done for WNV (Figure 1e and 1f), would offer further evidence for the generation of sfRNA in the SG and different release pathway.

We have added new supplementary figures showing correlations between gRNA and sfRNA within SG and saliva for ZIKV-inoculated Aedes (S11 Fig.). While the discrepancy between the correlations in SG and saliva is less obvious with micro-injected mosquitoes, there remains a lower correlation between gRNA and sfRNA in saliva than in SG ($R^2 = 0.24$ vs $R^2 = 0.72$). Inoculation results in higher infection levels which may affect the sfRNA/gRNA correlation in saliva.

These results are detailed in the result section:
l. 211-213: "As for WNV-infected Culex, ZIKV gRNA and sfRNA correlations were different between SG and saliva with a weaker correlation in saliva (S11a,b Fig.). »

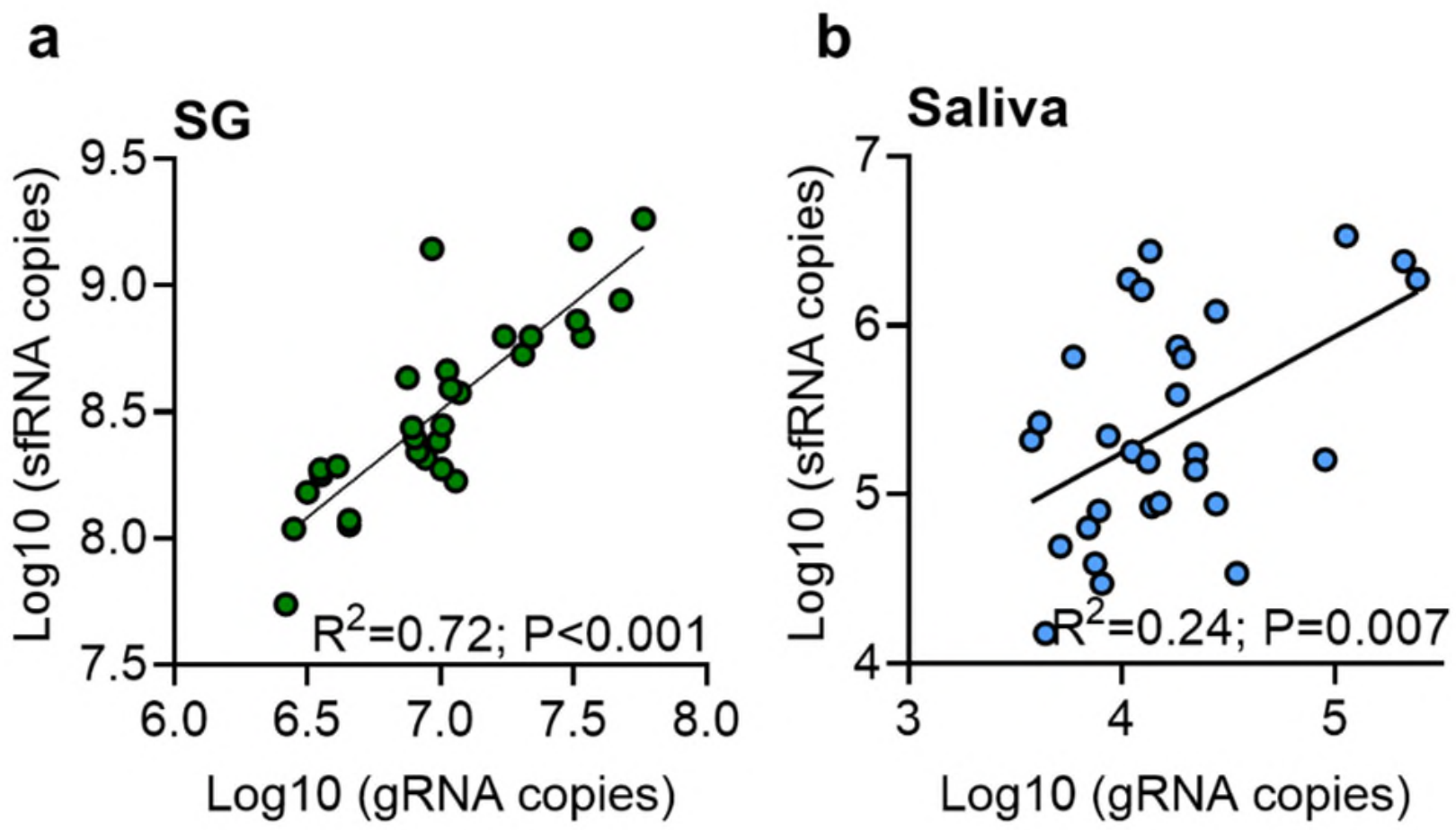

S11 Fig. (Related to Fig. 1) | a-b Correlations between gRNA and sfRNA copies within salivary glands (SG) and saliva from ZIKV-inoculated mosquitoes. Dots indicate SG and saliva repeats. P values were determined by correlation analysis.

10. Line 160: This conclusion is not supported by the data and should be revised. Thoracic inoculation bypasses but does not necessarily prevent midgut infection.

Agreed and we modified the sentence that now reads: (l. 172-174) "Collectively, these results indicate that sfRNA secretion is an intrinsic feature of salivary gland infection rather than dependent on the route of viral entry".

11. Figure 2: As suggested above in major point A, it may be challenging to definitively conclude that sfRNA and gRNA exist in separate particles due to potential differences in MNase sensitivity. The authors should consider revising the results section to reflect this limitation.

We agree with the comment and have generated new data showing the sensitivity to RNAse for a viral RNA control fragment (S12 Fig.) See also response to major comment A for more details. We have also tempered our conclusion by modifying the conclusion sentence as such:

l. 253-255: "Altogether, the analysis of saliva from two different mosquito genera infected with different orthoflaviviruses **suggest** the conservation of sfRNA secretion within salivary lipid vesicles across multiple orthoflaviviruses."

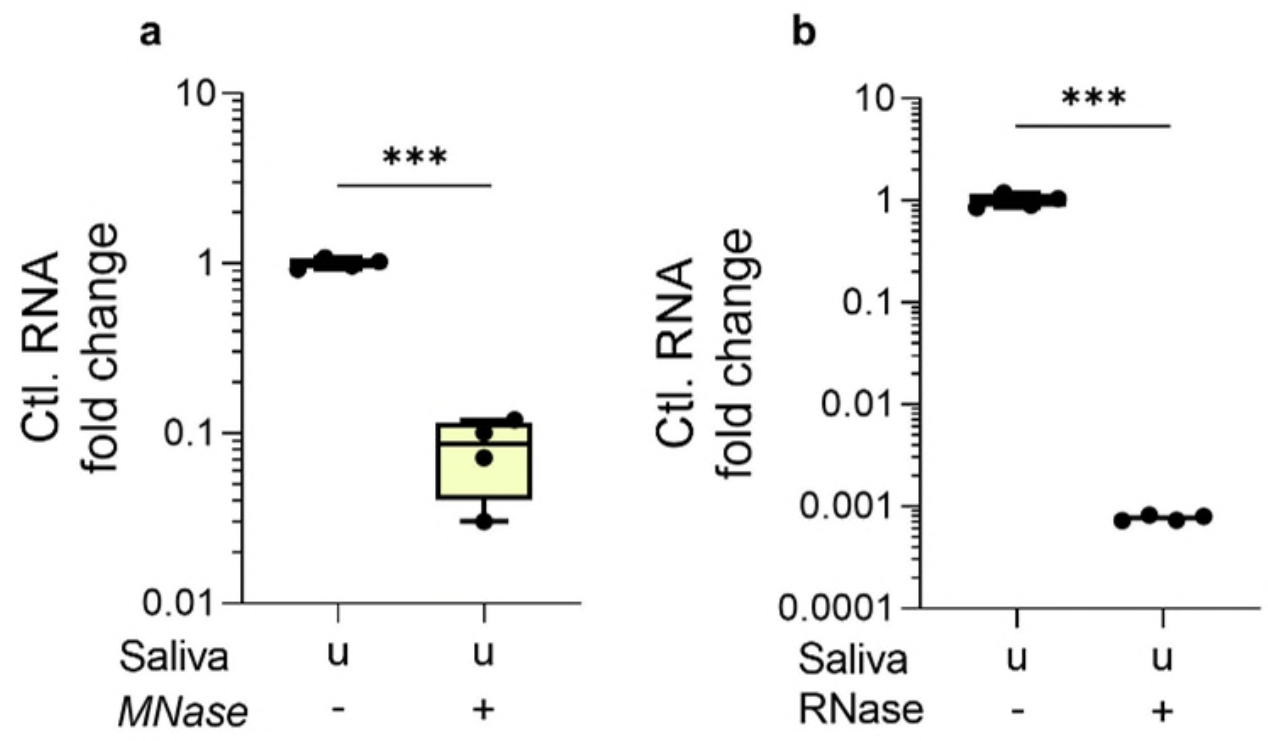

S12 Fig. (Related to Fig. 2) | a-b MNase and RNase degrade a viral RNA control. Boxplots indicate median ± lower and higher quartiles. N, 4. ***, $p < 0.001$ according to T-test.

12. Figure 3b and 3c: Please provide insight into why there is no observed increase in the measurements from 24 to 48 hours post infection.

There was no increase in infection levels between 24 and 48h because we normalized infection to L (low sfRNA concentration) within each time point. We thank the reviewer for pointing to this lack of clarity and have now explained in the legend that: "Infection levels were normalized to category L within time points."

13. Figure 3d and f: As suggested above in major point B, if gRNA is not indicative of saliva infectiousness, normalization of the sfRNA levels based on saliva infectiousness could provide a clearer analysis.

We now show that gRNA levels are indicative of infectiousness, by providing a strong correlation between gRNA and PFU in saliva (new S15a Fig.). To this end, we generated new saliva samples reaching 9 repeats to strengthen this correlation and justify normalizing infection levels by gRNA levels.

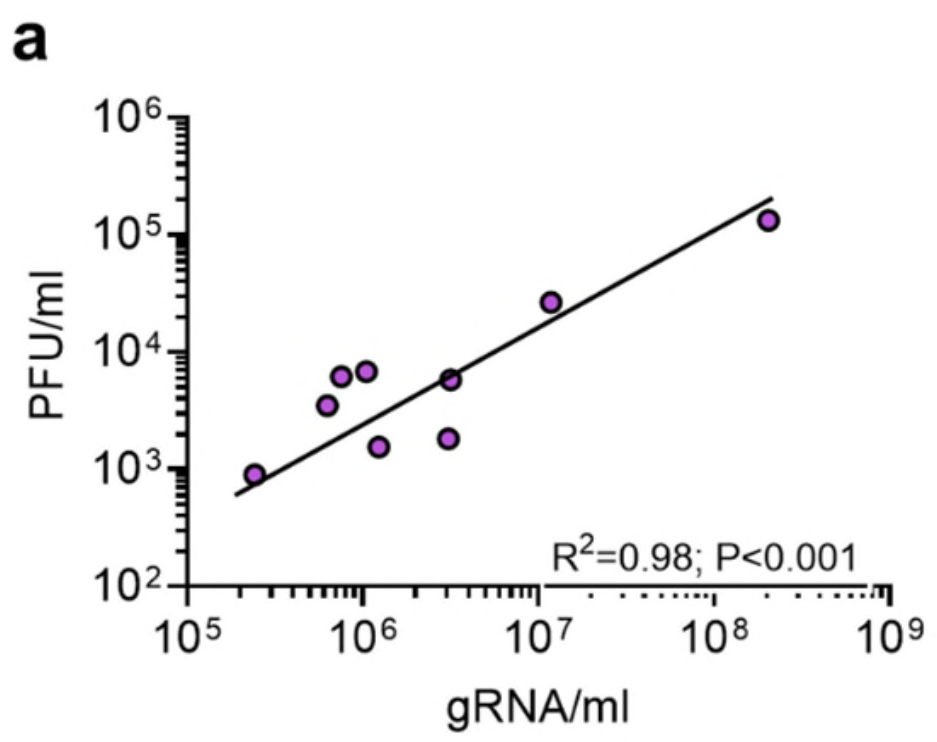

We added this information in the results section (l. 265-269):

“To support the normalization of saliva inoculum dose based on gRNA quantities, we showed that gRNA quantities were indicative of saliva infectiousness as measured by particle forming unit (PFU) (R^2 = 0.98; S15a Fig.) »

14. Figure 4a-d: Similar to Figure 3d and 3f, it would be more relevant to normalize sfRNA levels by saliva infectiousness.

As mentioned above, we now provide strong support for the normalization of infectiousness in terms of gRNA (new S15a Fig.). While we agree gRNA quantification represents a proxy for infectiousness, the lower volume (10µl) required for gRNA quantification as compared to PFU titration (at least 100 µl) allowed us to conserve sufficient saliva samples to perform the *in vitro* and *ex vivo* infection experiments.

15. To improve readability, it would be beneficial to standardize the labeling of the y-axis (e.g., using log10, 10x, or fold change) as well as the line scales consistently throughout the manuscript.

We have harmonized the labeling and axis scales across the figures by using a log-scale y-axis for gRNA copies, sfRNA copies, and fold changes. Nonetheless, we chose to retain different units for mosquito samples on the one hand and cell or explant samples on the other. In mosquito samples, gRNA and sfRNA levels are presented as absolute copy numbers per unit, as one mosquito, one salivary gland (SG), or one saliva sample was considered the normalizing unit. In contrast, for cell and skin explant experiments, gRNA levels were normalized to housekeeping genes to account for variation in cell number, and are therefore presented as fold changes.

Reviewer #2 (Remarks to the Author):

I co-reviewed this manuscript with one of the reviewers who provided the listed reports. This is part of the Nature Communications initiative to facilitate training in peer review and to provide appropriate

recognition for Early Career Researchers who co-review manuscripts.

We appreciate the reviewer's participation in the evaluation process, as well as their contributions and the time spent reviewing our work as part of this Nature Communications initiative.

Reviewer #3 (Remarks to the Author):

The article describes the role that sfRNA produced from the flavivirus 3'UTR contained in extracellular vesicles in mosquito saliva, plays in enhancing viral infection. The study reports the identification of sfRNA molecules being contained in EVs similar to a previous study by the authors. Authors employed various techniques to demonstrate and quantity sfRNA produced in culex and aedes mosquitoes saliva EVs for WNV and ZIKV respectively. They proceeded to show that increasing amounts of sfRNA-EVs in saliva increase early replication of the virus in both cell and skin models. They further added by showing that co-injection of WNV and sfRNA in mice increased disease severity and mortality compared to co-injection of WNV and control RNA. They then went on to show that addition of sfRNA had mixed results in inhibiting IFN response in different cell lines but was effective in the skin explants. Finally, authors concluded by showing that sfRNA could inhibit MDA5 signalling pathways as the mechanisms for inhibition.
The manuscript has a number of major deficiencies in methodologies (as listed below) leading to questioning of the main conclusions drawn from the results.

Major Concerns
1. There is no conclusive evidence of sfRNAs in mosquito saliva provided. Authors only show northern blot for sfRNA using total RNA extracted from whole mosquitoes. Authors should also provide northern blot of RNA from saliva with detectable sfRNA. Pulled saliva samples can be used to ensure sufficient amount sfRNA for northern blot detection.

We concur with the reviewer that direct detection of sfRNA in mosquito saliva is important to ascertain our discovery that sfRNA is secreted. We therefore followed the request and collected saliva from hundreds of WNV-infected Culex mosquitoes, pooled them and ultracentrifugated the solution to collect EVs. After RNA extraction, we performed NB as previously with whole mosquitoes. However, despite our repeated attempts, we could only get a very faint band, which is now presented in S6 Fig. and shown below.

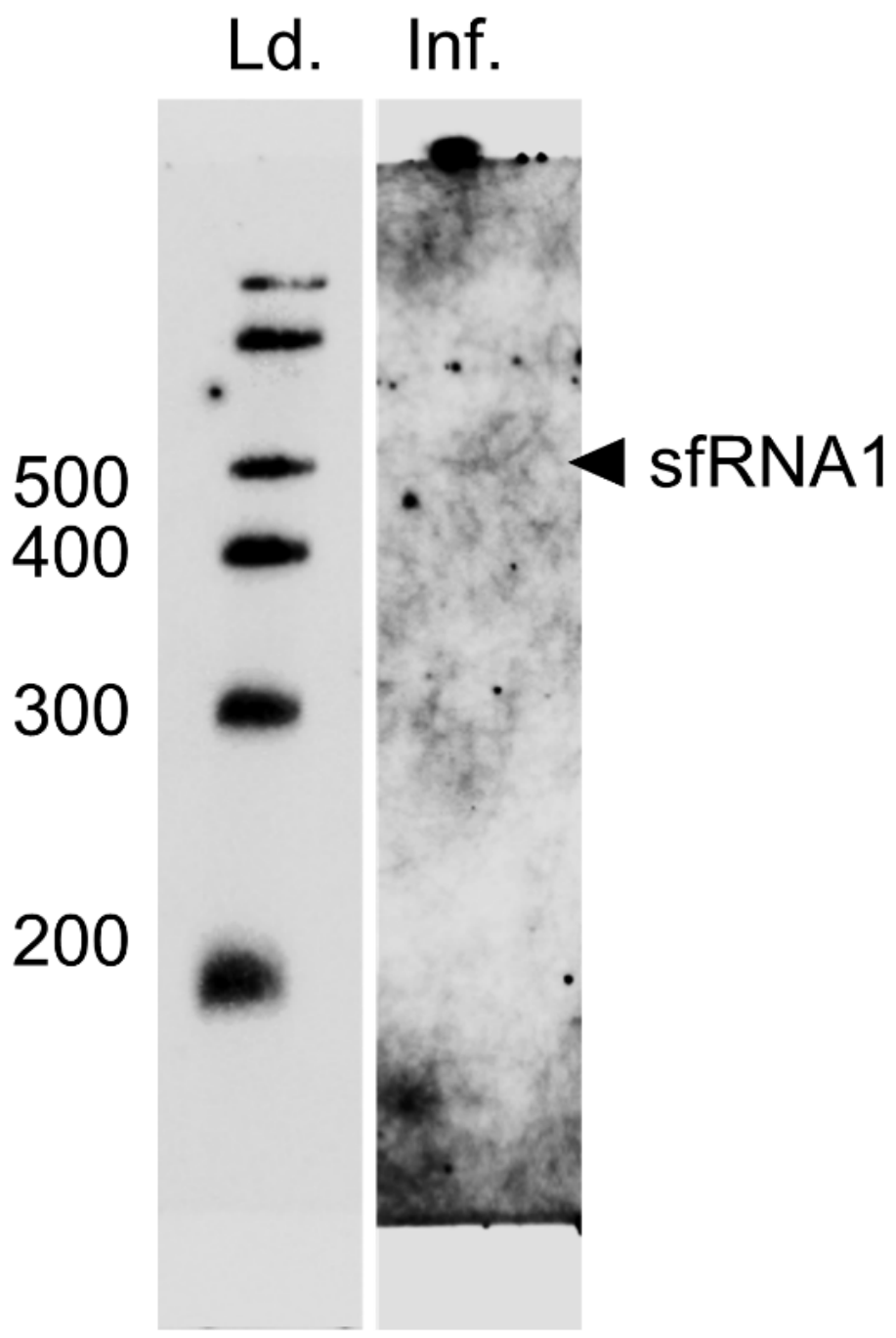

Although a band appeared at the expected size, we were not fully convinced by this result. We therefore made substantial efforts (hence the time for submitted the revision) to develop a novel molecular tool to directly detect sfRNA, as suggested by the reviewer. The results obtained with this new sfRNA detection method are considerably more robust (as detailed below). When combined with subtractive RT–qPCR detection/quantification, Northern blot analysis, and our previous detection of DENV sfRNA in *Aedes* saliva, these complementary approaches provide multiple independent lines of evidence supporting the secretion of sfRNA in mosquito saliva.

2. Quantification of sfRNA by comparative RT-qPCR with genomic viral RNA has significant flaws in its method. Authors have stated in their previous paper that this may overestimate the sfRNA concentrations. It does not allow for differences in processing of genomic RNA to sfRNA or efficiency in RT and PCR. Direct quantification methods which quantify sfRNA without a need to quantify viral genomic RNA should be established similar to those found in studies involving small non-coding RNA. This may involve ligating adapter to 5' end of sfRNA following by RT-PCR with primers complementary of 3' end of sfRNA and to the adapter-5'end of sfRNA junction.

We fully agree with the reviewers that direct detection of sfRNA in mosquito saliva is critical. Because conventional Northern blotting lacks the sensitivity for low RNA copy numbers in individual saliva samples, we invested substantial effort to design and validate a sensitive molecular assay and ended up designing a splinted-ligation RT–PCR, a molecular assay used for detecting RNA decay intermediates. This novel approach now unequivocally demonstrates the presence of sfRNA in saliva from infected mosquitoes.

As suggested by the reviewer, we started our journey by getting inspiration from established miRNA quantification techniques that use stem-loop primers. In these approaches, a stem-loop primer specifically anneals to the 3’ end of the miRNA and serves as the reverse primer for reverse transcription (RT). However, applying this method directly to sfRNA would result in the reverse transcription of both sfRNA and genomic RNA (gRNA), thereby compromising specificity, as we with subtractive RT-qPCR method.

To overcome this, we attempted an alternative strategy inspired by both miRNA and G-quadruplex quantification [5]. The method consists of several key steps. First, reverse transcription was performed using a primer annealing within the 3’ UTR and the superscript III RT enzyme because it does not add nucleotides to the 5’ end. The RT generated cDNA from both sfRNA and gRNA, but importantly produced a sfRNA cDNA 3’end that corresponds to the 5’ end of sfRNA. Second, the RT primer was then enzymatically degraded to prevent interference with downstream amplification. Third, a stem-loop primer was designed to specifically anneal to the 3’ end of the sfRNA cDNA (which corresponds to the 5’ end of the original sfRNA molecule), thereby excluding gRNA-derived cDNA. Fourth, this stem-loop primer was then ligated to the sfRNA cDNA. Fifth, qPCR amplification was carried out using a reverse primer within the sfRNA cDNA (to avoid amplification of the stem-loop alone) and a forward primer binding to the loop region of the stem-loop structure. Finally, sfRNA was quantified using a TaqMan probe that binds to the junction between the sfRNA and the stem-loop sequence, ensuring specificity for sfRNA.

We initially validated sfRNA detection using a synthetic fragment corresponding to the 5’ end of sfRNA. However, upon dilution of this synthetic template, a second band was amplified, indicating competition between the sfRNA target and another fragment, which we believe corresponds to the stem–loop primer. As a result, this assay displayed a relatively high detection threshold and, as expected, failed to detect sfRNA of the expected size when applied to mosquito saliva.

As an alternative approach, we developed a direct sfRNA detection assay inspired by splinted-ligation RT–PCR (SL-RT-PCR), a method commonly used to detect mRNA decay fragments that also carry a mono-phosphorylated 5’ end. In this assay, a DNA splint complementary to both an anchor RNA and the sfRNA sequence brings the two RNA molecules into proximity, allowing T4 DNA ligase to ligate the monophos-phorylated 5’ end of sfRNA to the anchor RNA. Following degradation of the splint DNA, the resulting chimeric RNA is specifically amplified by RT–PCR using primers targeting the anchor RNA and sfRNA sequences.

After validating the SL-RT-PCR assay using the synthetic 5’ sfRNA fragment and appropriate controls, we are very happy to report that we successfully detected sfRNA in saliva from WNV-infected mosquitoes. These new results are presented in Fig. 1j, as presented below.

We thank the reviewer for prompting us to improve the methodology for sfRNA detection. This effort led to the development of a novel detection approach that can be readily adopted by laboratories studying sfRNA and represents a significant methodological improvement.

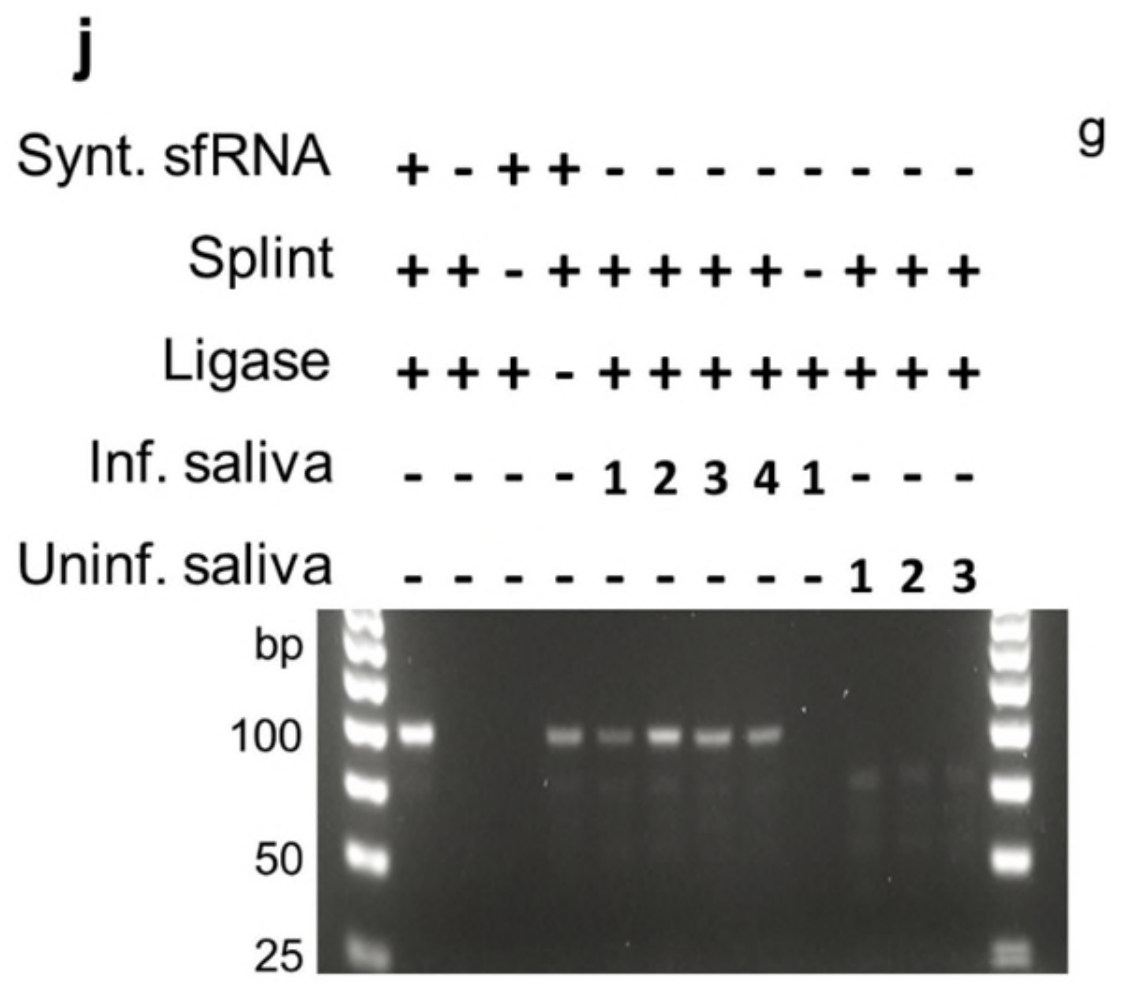

3. Figure 4A uses saliva extracted from mosquitos where differences in viral inoculation may occur (as stated by authors). Therefore, quantities of infectious virus (i.e. viral titres) should also be included for analysis and to be used for normalisation.

We concur that the normalization of saliva infectiousness is crucial for the interpretation of our functional experiments. We now present data indicating that gRNA levels are a reliable measure of infectiousness, as determined by plaque-forming unit (PFU) assays (S15a Fig.). In this new figure panel, we have combined data from previous and new saliva samples, totaling nine samples, which significantly supports the positive correlation between gRNA levels and PFU in saliva, and therefore the use of gRNA levels as a proxy for infectiousness.

While we acknowledge that gRNA quantification serves as a proxy for infectiousness, its lower volume requirement (10 µL compared to ≥ 100 µL for PFU titration) enabled us to conserve sufficient saliva samples for in vitro and ex vivo infection experiments.

To justify the use of gRNA to normalize inoculum, we added the new information in the results section (l. 265-268): "To support the normalization of saliva inoculum dose based on gRNA quantities, we showed that gRNA quantities were proportional to saliva infectiousness as measured by particle forming unit (PFU) ($R^2 = 0.98$; $p < 0.001$; S15a Fig.)".

a

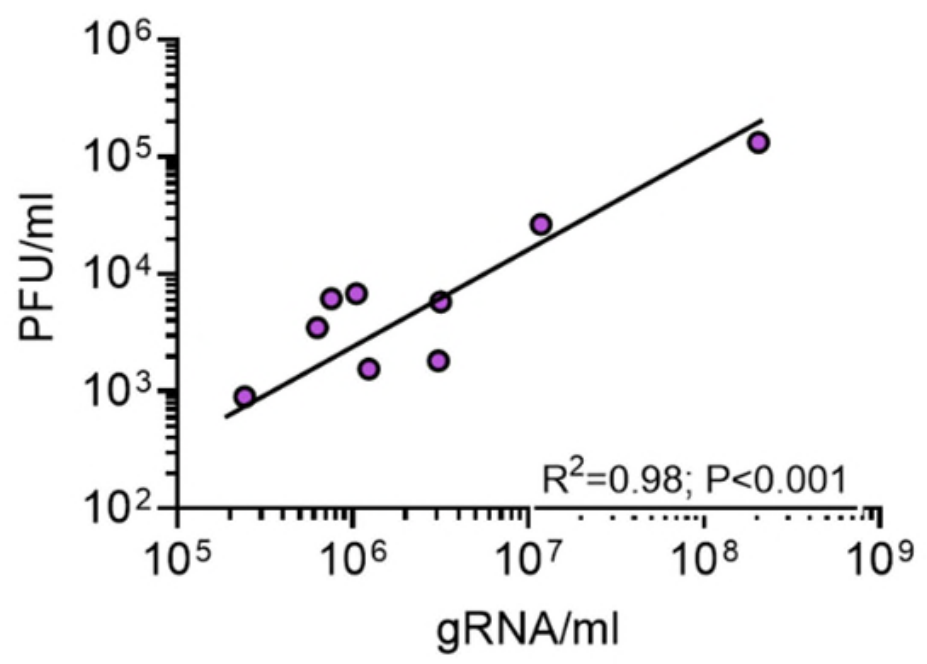

4. In Fig 4e-t and Fig 5 b-d authors used extremely low MOI (MOI=0.005) of infection with sfRNA-producing virus and transfected extremely high copies of sfRNA (10e10 copies) resulting in inconclusive results because very few cells would be infected and mount IFN response. Rescue of IFN response by sfRNA using higher MOI infection with sfRNA deficient viruses would provide more appropriate analysis because this will allow to standardise cell response to the virus in the absence of sfRNA and adequately access the role of exogenously added sfRNA on these responses.

We initially selected a multiplicity of infection (MOI) of 0.005 to achieve an sfRNA:gRNA ratio approximating that found in mosquito saliva (=800), thereby ensuring physiologically relevant experimental conditions.

We apologize for the lack of clarity regarding our rationale and have now included a detailed breakdown of the calculation in the results section:
L. 279-286: "we transfected Huh7 cells with *in vitro*-transcribed sfRNA shortly before WNV infection (Fig. 3g). Intracellular levels of sfRNA or a same-size viral RNA control were comparable after transfection, averaging ~10^8 copies per 250,000 cells (S16a Fig.). To achieve a sfRNA:gRNA ratio approximating that observed in mosquito saliva (Fig. 1d), we employed a low inoculum (MOI 0.005), corresponding to 1,250 PFU. Based on a gRNA:PFU ratio of 100 - commonly observed in virus preparations from mosquito cells [47] - this inoculum was estimated to contain 125,000 gRNA copies, resulting in a high sfRNA:gRNA ratio of 800."

This sfRNA:gRNA ratio is in the same order of magnitude as the average sfRNA:gRNA ratio measured in mosquito saliva (~3,379; Fig. 1d), and importantly, lower than the salivary ratio. As such, our experimental design avoids artificially inflating the functional impact of sfRNA, ensuring a conservative assessment of its role in saliva-mediated viral transmission.

Nonetheless, to answer the valid reviewer's comment about potential under activation of IFN with low MOI, we repeated the experiments with a higher MOI of 0.5 in the three cell types (i.e., Huh7, HFF1 and U937) and quantified gRNA, IFNb, CXCL10, MX1 and IFI6. The new S17 Fig confirmed the infection increase upon sfRNA transfection in all three cell lines and actually provided stronger support for the

inhibition of IFN response in the three cell lines, potentially as a result of higher IFN induction, as suggested by the reviewer.

These new interesting datasets were integrated by significantly re-writing the result section:
For infection; l. 292-297: “To rule out biases associated with low MOI - where few cells are initially infected – and the use of high sfRNA:gRNA ratio, we repeated the experiments with a higher MOI of 0.5 in all three cell types, resulting in an estimated very low sfRNA:gRNA ratio of 8. Notably, the pro-viral function of sfRNA was maintained under these higher inoculum conditions (S17a-c Fig.).”
For the immune response; l. 331-339 : “In Huh7 cells, sfRNA transfection inhibited the expression of IFN-β and IFI6 at 24 and 48 hpi for both inocula, while CXCL10 and MX1 expressions were diminished at 48 hpi with the low inoculum only (Fig. 4e-h; S17d-g Fig.). In HFF1 cells, under low MOI infection, sfRNA strongly suppressed IFN-β expression at 24 hpi and reduced CXCL10 expression at both 24 and 48 hpi (Fig. 4i-l). Under high MOI conditions, all four markers of IFN respone were downregulated at 48 hpi (S17h-k Fig). In U937 cells, sfRNA transfection under low MOI did not inhibit the expression of the IFN-related genes (Fig. 4m-p); however, all four IFN markers were downregulated at 48 hpi, with IFN-β also reduced at 24 hpi under high MOI conditions (S17l-o Fig.).”

Altogether, we thank the reviewer for questioning the low MOI as this helped us to improve clarity on our experimental design and provide additional data that strengthen our conclusion.

Regarding the use of sfRNA-deficient viruses, we respectfully disagree as this approach may bias the evaluation of our hypothesis. Our objective is to evaluate the function of early presence of sfRNA. The absence of sfRNA production following infection could artificially inflate the effect of input sfRNA and alter both infection level and IFN responses. While we acknowledge that sfRNA-deficient viruses are valuable tools to dissect sfRNA-related mechanisms, in our specific context we consider wild-type viruses to be more appropriate, as they better mimic the natural scenario of early sfRNA delivery in conjunction with incipient sfRNA production.

Also authors need to provide an evidence that sfRNA is actually being transfected and present in transfected cells, e.g. transfect FAM-labelled sfRNA.

Concerning the validation of sfRNA delivered intracellularly, we have provided a direct quantification of intracellular sfRNA by using RT-qPCR in all three cell lines used in the functional assay (S13a-c Fig.), as detailed .
l. 280-281: “Intracellular levels of sfRNA or a size-matched viral RNA control were comparable after transfection, averaging ~10^8 copies per 250,000 cells (S16a Fig.).”

5. Previous studies have implicated STAT1 inhibition by sfRNA as an inhibitor of IFN pathways. Why did authors not investigate this mechanism as well?

We thank the reviewer for raising this important hypothesis, which we had asked ourselves. A previous study interestingly showed that ZIKV sfRNA stabilizes NS5, enhancing NS5 inhibitory effect on STAT1 and thereby reducing IFN signaling [7]. While we considered testing how STAT1 depletion might influence the function of delivered sfRNA, we opted for a stepwise approach that started upstream of STAT1 by examining RIG-I and MDA5 [8]. After determining that MDA5 depletion abolished the infection enhancement mediated by sfRNA delivery, we chose not to pursue STAT1 depletion. Nevertheless, we acknowledge that delivered sfRNA may also interact with NS5 or potentially modulate other immune components, as discussed in Slonchak et al., 2022 (l. 435-437).

Bibliography

1. Pijlman, G. P. *et al.* A highly structured, nuclease-resistant, noncoding RNA produced by flaviviruses is required for pathogenicity. *Cell Host & Microbe* **4**, 579–591 (2008).
2. Hoek, T. A. *et al.* Single-Molecule Imaging Uncovers Rules Governing Nonsense-Mediated mRNA Decay. *Mol Cell* **75**, 324-339.e11 (2019).
3. Syenina, A. *et al.* Positive epistasis between viral polymerase and the 3′ untranslated region of its genome reveals the epidemiologic fitness of dengue virus. *PNAS* (2020) doi:10.1073/pnas.1919287117.
4. Pallarés, H. M. *et al.* Zika Virus Subgenomic Flavivirus RNA Generation Requires Cooperativity between Duplicated RNA Structures That Are Essential for Productive Infection in Human Cells. *J Virol* **94**, e00343-20, /jvi/94/18/JVI.00343-20.atom (2020).
5. Kwok, C. K. & Balasubramanian, S. Targeted Detection of G-Quadruplexes in Cellular RNAs. *Angew Chem Int Ed Engl* **54**, 6751–6754 (2015).
6. Vial, T. *et al.* Mosquito metabolomics reveal that dengue virus replication requires phospholipid reconfiguration via the remodeling cycle. *Proc Natl Acad Sci USA* 202015095 (2020) doi:10.1073/pnas.2015095117.
7. Slonchak, A. *et al.* Zika virus noncoding RNA cooperates with the viral protein NS5 to inhibit STAT1 phosphorylation and facilitate viral pathogenesis. *Sci Adv* **8**, eadd8095 (2022).

8. Onomoto, K., Onoguchi, K. & Yoneyama, M. Regulation of RIG-I-like receptor-mediated signaling: interaction between host and viral factors. *Cell Mol Immunol* **18**, 539–555 (2021).

Dear Reviewers,

I am thankful for your time and engagement to improve the manuscript robustness.

While we acknowledge that reviewer 1 is satisfied with our initial revision, we have now addressed the remaining comments from the combined revision of reviewer 2 and 3 in a point by point format written in blue.

I am convinced that thanks to the reviewers' requests the second revision provides very strong support for our conclusion.

Best wishes

Julien Pompon.

REVIEWER COMMENTS

Reviewer #1 (Remarks to the Author):

The authors have addressed all previously raised concerns in a convincing manner. Their thorough revision has substantially improved the quality of the manuscript.

Reviewer #2 (Remarks to the Author):

I co-reviewed this manuscript with one of the reviewers who provided the listed reports. This is part of the Nature Communications initiative to facilitate training in peer review and to provide appropriate recognition for Early Career Researchers who co-review manuscripts.

Reviewer #3 (Remarks to the Author):

Major concerns 1 and 2 – evidence for sfRNA in saliva and quantification of sfRNA.
These are still the major issues with this manuscript.

Authors provided very poor-quality Northern blot presumed to show sfRNA in a pooled saliva sample from 100 mosquitoes. There is no distinct band in the blot that would convincingly demonstrate presence of sfRNA, which authors themselves admit in their response.

We agree with the reviewer and were honest in the text of the first revision. The band is very faint and not strongly convincing. The inherent limits of minute volumes of saliva and the low resolution of Northern blot are probably the causes of this unsatisfactory detection.

The authors then went to test several other methods for sfRNA detection with most of them failing.

However, one of the methods, based on splinted-ligation RT-PCR appeared to work (new panel J in the figure). The addition of panel J goes some way to alleviating concerns about detecting sfRNA directly, however authors have not addressed the concerns over quantification. There are limited details about the protocol, including amount of input RNA used in the reverse transcription, as well as the number of cycles used in the PCR.

We want to stress that with the development of the SL-RT-PCR, we provide the first direct molecular detection of sfRNA, overpassing technical limits that restricted the field since the discovery of sfRNA in 2008 (Pijlman et al; 2008. CHM).

We apologize for missing to mention the input RNA volume and have now added this information in the methods (l. 630).
Nonetheless, we want to highlight that we had already mentioned the details for the PCR amplification as detailed below:
l. 640-645: "RT-PCR amplification was performed using one-step RT-PCR (Bio-Rad) and 300 nM of RNA anchor-matching forward (5'-GCTGATGGCGATGAATGAACACTGC-3') and sfRNA-matching reverse (5'-GCAGGCAGCACCGTCTACT-3') primers in total volume of 10 µl in LightCycler 96 (Roche) with the following thermal profile: 50°C for 10 min, 95°C for 1 min, followed by 40 cycles of 95°C for 10 sec, 60°C for 25 sec."

Additionally, the amplification in the sample where key enzyme, ligase, was absent raises concerns about non-specific amplification. An additional control using synthetic RNA with the 5' end further upstream (as to simulate genomic RNA) would also improve the validity of the experiment. Were the products sequenced to confirm generation of the appropriate fragments?

We thank the reviewer for suggesting this appropriate control. As suggested, we produced a new control of monophosphorylated RNA encompassing the sfRNA sequence control plus 10 nucleotides upstream of the 5'end. Confirming the specificity of the SL-RT-PCR method, the new S7 Fig. shows that there was no amplification at the expected size.
These new results are presented in the results:
l. 190-193: "We further validated the specificity of the SL-RT-PCR assay by showing a lack of amplification when the synthetic monophosphorylated template encompassed the sfRNA target plus 10 nucleotides upstream of the 5'-end (S7 Fig.)."

Although the authors have added this figure, they have not used this method to quantify the virus and still rely of the subtractive PCR methods in the original manuscript. Why did authors not use this direct quantification method to quantify sfRNA in their saliva pools?

We did not quantify the sfRNA because an absolute standard curve could not be established. Although the sfRNA control fragment was provided by the manufacturer at a defined initial concentration, it subsequently underwent monophosphorylation and purification in our laboratory. Each of these steps resulted in material loss, yielding a monophosphorylated sfRNA control fragment of unknown final concentration. Consequently, this product could not be used to generate a reliable absolute standard curve.

Variation in band intensity of direct sfRNA RT-PCR in the new panel J compared to the original estimates (Line 191) suggests an error in one of these techniques.

We had indeed inverted the order of samples. The text is now modified as such:
l. 194: "containing 9.7 x 10^4, 1.45 x 10^6, 4.4 x 10^5 and 2.25 x 10^5 sfRNA copies for samples 1-4, respectively"

There is now an apparent correlation between quantities obtained with the subtractive RT-qPCR and band intensity for the SL-RT-PCR. We have added this information in the results section:
l. 197-198: "Notably, band intensity from the SL-RT-PCR correlated with sfRNA quantities determined with subtractive RT-qPCR."

Major concern 4 – use of low MOI and detection of transfected sfRNA

MOI - the suggested to be used higher MOI emulates similar results. However, It doesn't appear that virus gRNA changes much in the higher MOI infections, did the authors do virus quantification?

In both Fig. 3h-j and S17a-c Fig., viral load was quantified in terms of gRNA fold change normalized to four different housekeeping genes (RPL13A, ACTB, B2M, and GAPDH) relative to ctl RNA transfection at 24h. As the variation in absolute gRNA is not presented, this may have confused the reviewer.

sfRNA transfection control - The RT-PCR of the transfected control RNA and sfRNAs is adequate to support this but a negative control would add greater clarity. Authors could test cells transfected with control RNA using sfRNA-specific primers and vice versa as negative controls.

As requested, we have performed the control experiments. We performed RT-qPCR using sfRNA-targeting primers directly on the control RNA and sfRNA, and vice versa. We reasoned that it would be more stringent to test the specificity on known concentration of templates, instead of testing it on cells that were transfected. The results are presented below in panel A for sfRNA/3'UTR primers and in panel B for control RNA primers each applied on sfRNA template and control RNA template, and water as negative control.

For sfRNA primers, we only detected sfRNA templates and no amplification occurred when control RNA templates or water was added.

For control RNA primers, we quantified control RNA templates with a melting temperature at 80.5°C. However, we also had an amplification with either sfRNA templates or water with a melting temperature at 78°C, indicating primer dimers. In our careful analysis of the qPCR results, we did not account for amplification with a different melting temperature than 80.5°C. Competition between control RNA target and primer dimer amplification may occur, but we think it to be minimal given that with concentration of control RNA templates around what we quantified in transfected samples there was no shoulder peak for the melting curve – see melting curve in panel B for control RNA 10^8 and 10^7. Control RNA and sfRNA were present at a mean of 10^8 copies in the three different cells we tested (S17 Fig.).

Additionally, control RNA and sfRNA transfections were done separately, precluding the cross detection with the qPCR.

A. sfRNA/3'UTR primers

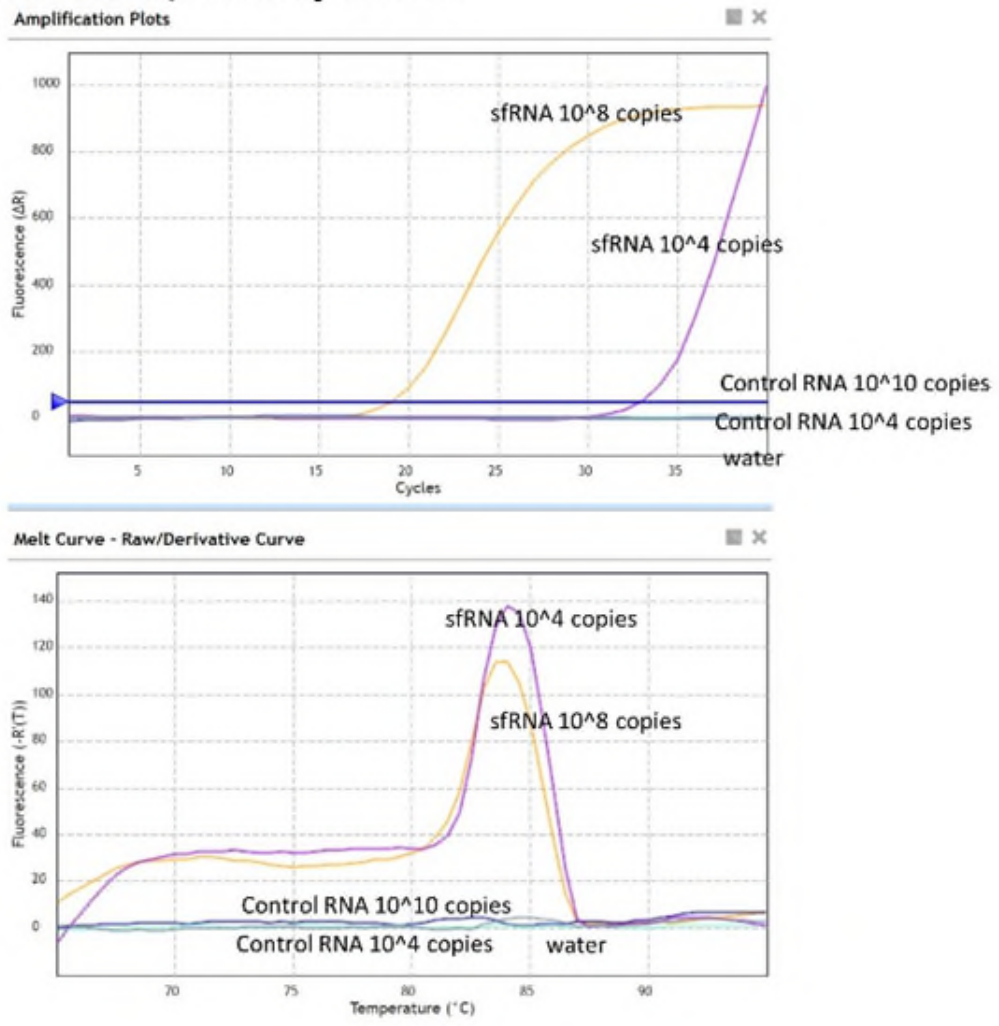

Well	Standards (copies)		Cq	Tm
A1	sfRNA	1,00E+08	19,15	84
A2	control RNA	1,00E+08	No Cq	93
F1	sfRNA	1,00E+04	33,07	84
F2	control RNA	1,00E+04	No Cq	85
B7	Neg CTRL	water	No Cq	86

B. Control RNA primers

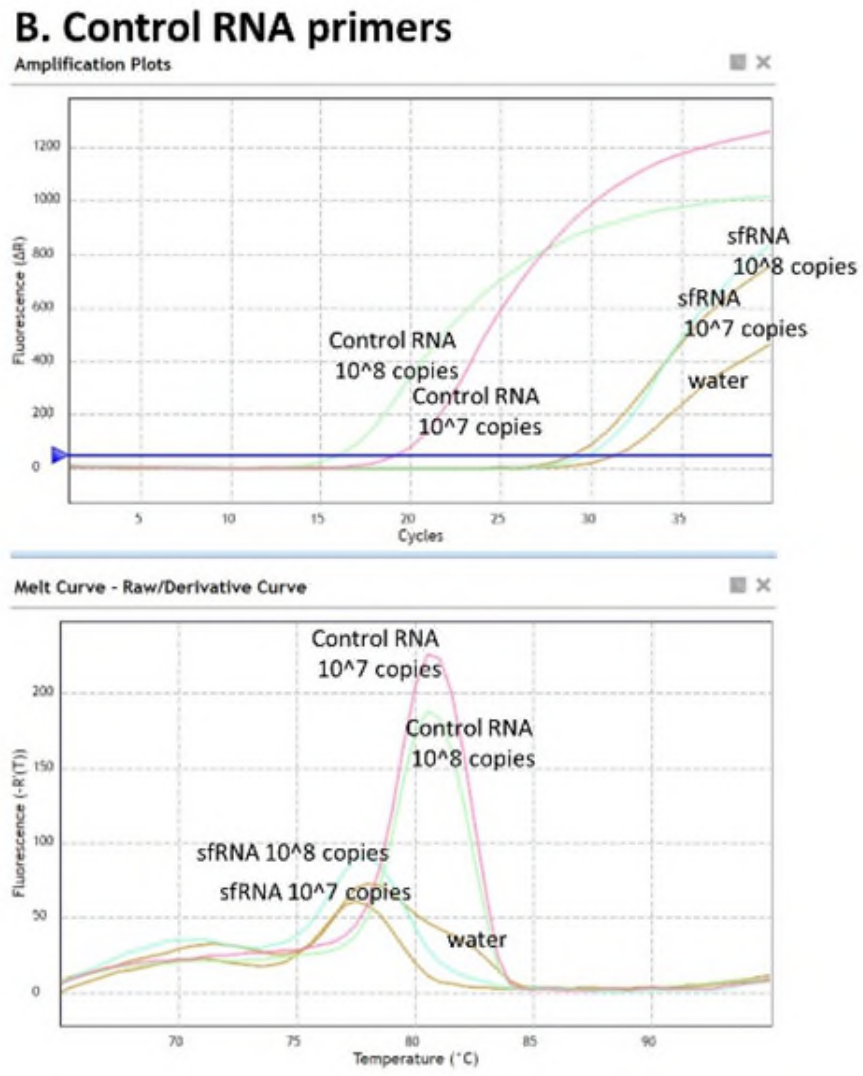

Well	Standards (copies)		Cq	Tm
B3	sfRNA	1,00E+08	29,15	78
B4	control RNA	1,00E+08	16,11	80,5
C3	sfRNA	1,00E+07	29,94	78
C4	control RNA	1,00E+07	19,31	80,5
A7	Neg CTRL	water	31,51	77,5

Point by point response to the reviewers' comment in blue.

Reviewer #3 (Remarks to the Author):

Authors provided adequate response and data for quantification of sfRNA, however the Northen blot detection of sfRNAs and our request for negative controls in sfRNA-transfected cells were not adequately addressed.

We thank the reviewer for acknowledging the adequacy of our response and quantification of sfRNA.

Northern bolts.
Bands labelled as sfRNA1 in saliva samples from WNV-infected mosquitoes in Fig S6 and in whole mosquito sample from ZIKV-infected mosquitoes in Figs S8 are still not convincing at all. These should be ether redone with different detection methods (as for example in Pallares et al 2020 and Slonchak et al 2020) to convincingly show detection of sfRNAs by Northern blots or removed from the paper with claims on detection of sfRNA by Northern blot also removed from the text.

As suggested by the editor, we added a comment to the manuscript acknowledging the limitations of the assays.

Negative sfRNA transfection controls.
While authors performed additional experiment to include amplification of in vitro transcribed sfRNA with control RNA primers and in vitro transcribed control RNA with sfRNA primers, no data on requested transfection controls for the uptake of sfRNA and detection with control primers as well uptake of control RNA and detection with sfRNA primers were presented. This was requested to adequately demonstrate consistent uptake of the RNAs by the cells and their specific detection in transfected cell environment. This would increase confidence in the specificity of sfRNA detection in transfected cells by RT-PCR.

We have performed the requested additional experiments consisting of (C) testing sfRNA/3'UTR primers on Aag2 cells transfected with RNA control fragments and (D) RNA control primers on Aag2 cells transfect ed with RNA control fragments. We also include our previous experiments directly testing (A) sfRNA/3'UTR primers on a controlled quantity of RNA control and (B) RNA control primers on a controlled quantity of sfRNA copies.

As seen in the qPCR plots below, sfRNA/3'UTR primers generate some low amplification (Cq>35) in RNA control-transfected Aag2 cells (C) with a Tm at 75.5°C. In the negative control we included, consisting only of the qPCR mix, we also observe this amplification with a Tm at 75.5°C, indicating that this amplification corresponds to primer dimers. In our other experiment where we tested sfRNA/3'UTR primers on a known quantity of in vitro-transcribed sfRNA (A), we amplified sfRNA with a Tm at 84°C, while we did no observe amplification when testing these primers on RNA control fragments. Altogether, these results show that sfRNA/3'UTR primers specifically amplify sfRNA and could in absence of sfRNA target produce primer dim ers at a different Tm with a low Cq. When analyzing our qPCR, we therefore excluded amplification above than a Cq of 35 with a Tm different from 84°C, as in good lab practices.

With RNA control primers, we reported similar observations. RNA control primers used in qPCR with sfRNA-transfected Aag2 cells or negative control (water in qPCR mix) did produce a low amplification (Cq > 29) with a Tm at 78°C (D). However, when amplifying a controlled quantity of RNA control copies, the amplified fragment had a Tm at 80.5°C and we did not observe a melting peak at 78°C (B). Additionally, when quantifying a controlled concentration of sfRNA, RNA control primers only produced a melting peak at 78°C (B). Altogether, these results show that RNA control primers are specific to RNA control fragments, not amplifying sfRNA, but could produce primer dimers when the RNA control target is absent. When analyzing our qPCR results, we excluded Cq higher than 29 and amplification with a Tm different than 80.5°C.

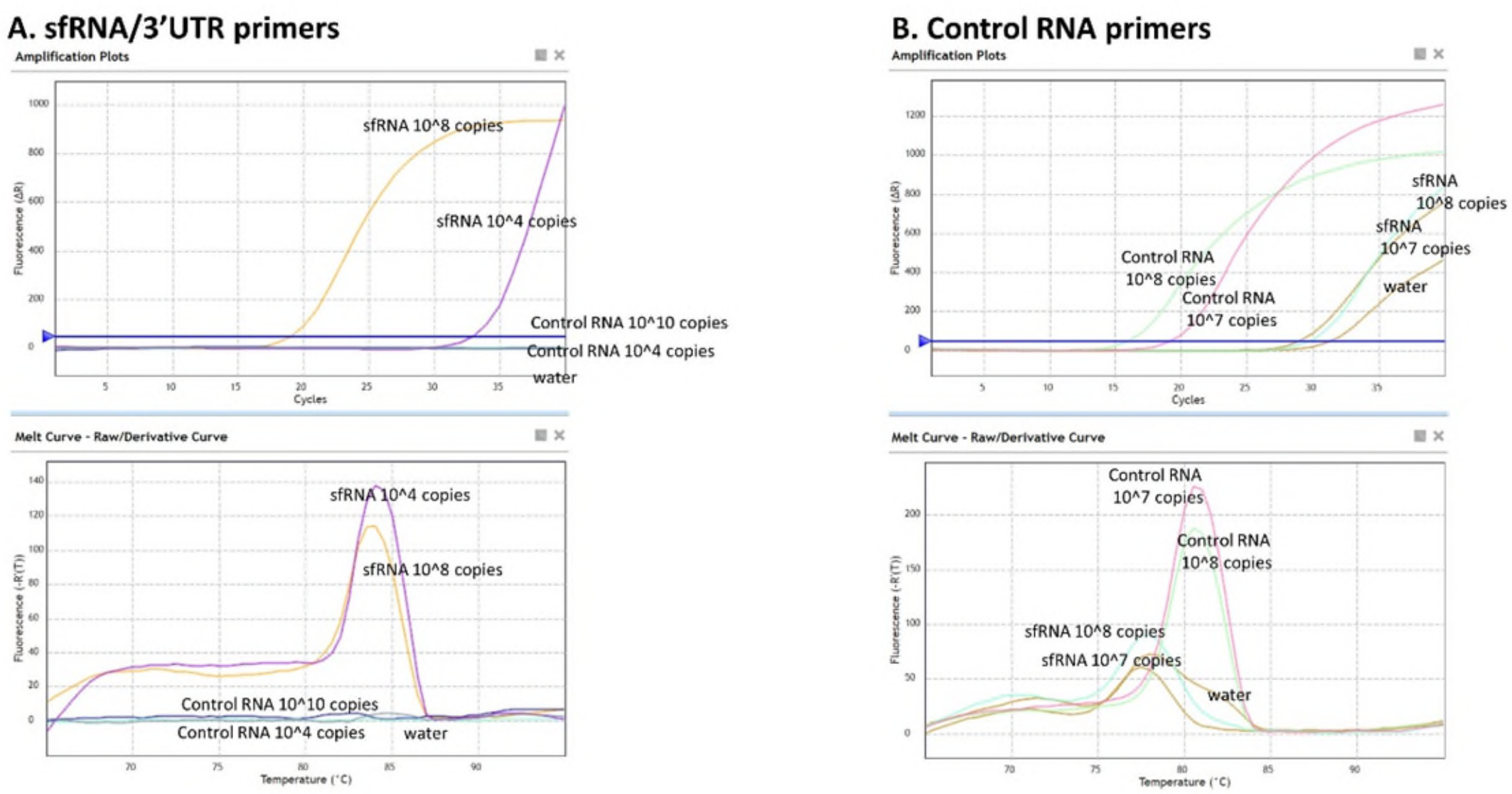

Well	Standards (copies)		Cq	Tm
A1	sfRNA	1,00E+08	19,15	84
A2	control RNA	1,00E+08	No Cq	93
F1	sfRNA	1,00E+04	33,07	84
F2	control RNA	1,00E+04	No Cq	85
B7	Neg CTRL	water	No Cq	86

Well	Standards (copies)		Cq	Tm
B3	sfRNA	1,00E+08	29,15	78
B4	control RNA	1,00E+08	16,11	80,5
C3	sfRNA	1,00E+07	29,94	78
C4	control RNA	1,00E+07	19,31	80,5
A7	Neg CTRL	water	31,51	77,5

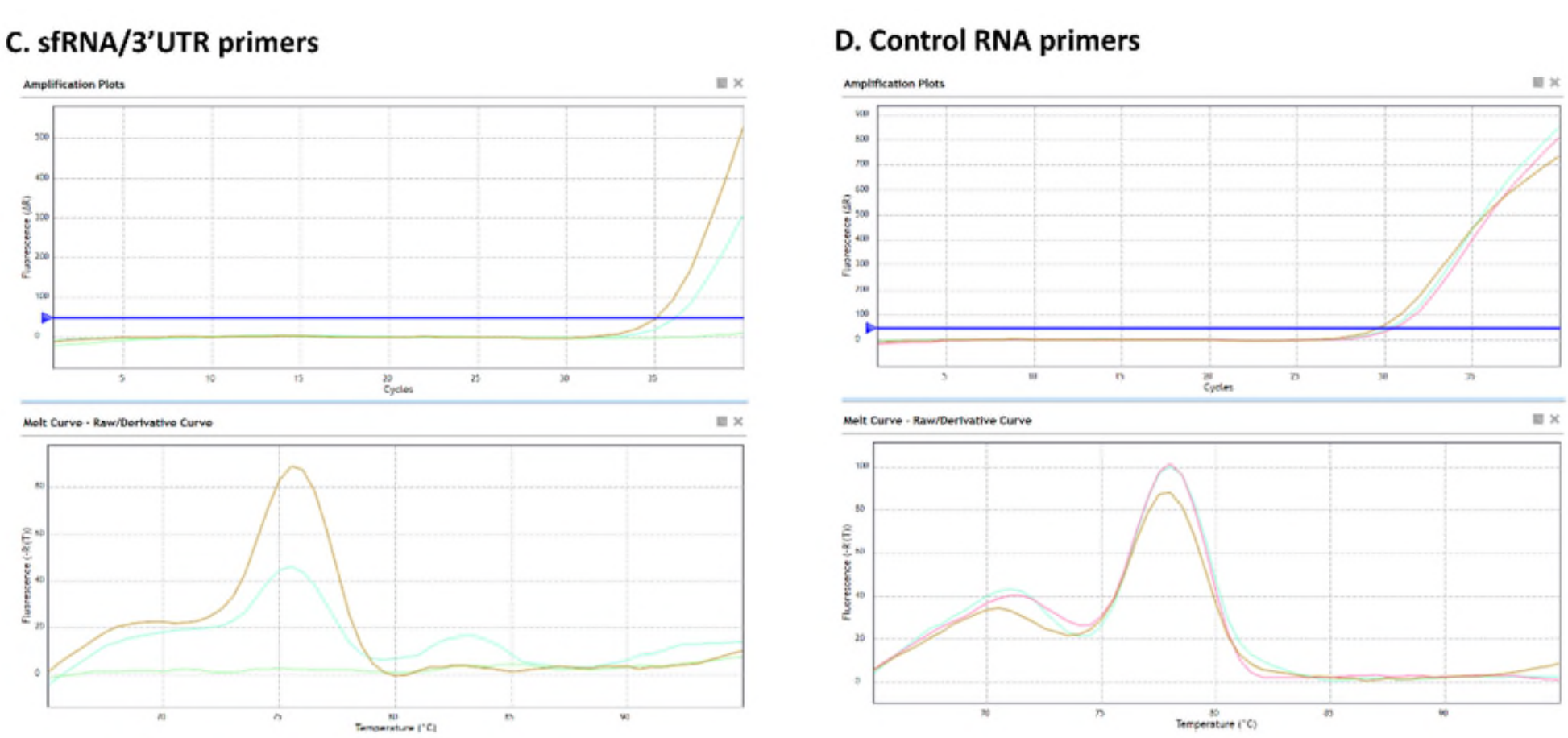

A11	Aag2 #1	36,27	75,50
A12	Aag2 #2	No Cq	95,00
D11	Neg	35,15	75,50

E11	Aag2 #1	30,44	78,00
E12	Aag2 #2	30,69	78,00
H11	Neg	29,80	78,00
