# Supplementary material for: Multiple orthoflaviviruses secrete sfRNA in mosquito saliva to promote transmission by inhibiting MDA5-mediated early interferon response
Source: Nat Commun. 2026 Aug 29;17:10323. doi: 10.1038/s41467-026-77288-4 (PMC13623828; doi:10.1038/s41467-026-77288-4)

Source Data for images

Fig. 1a

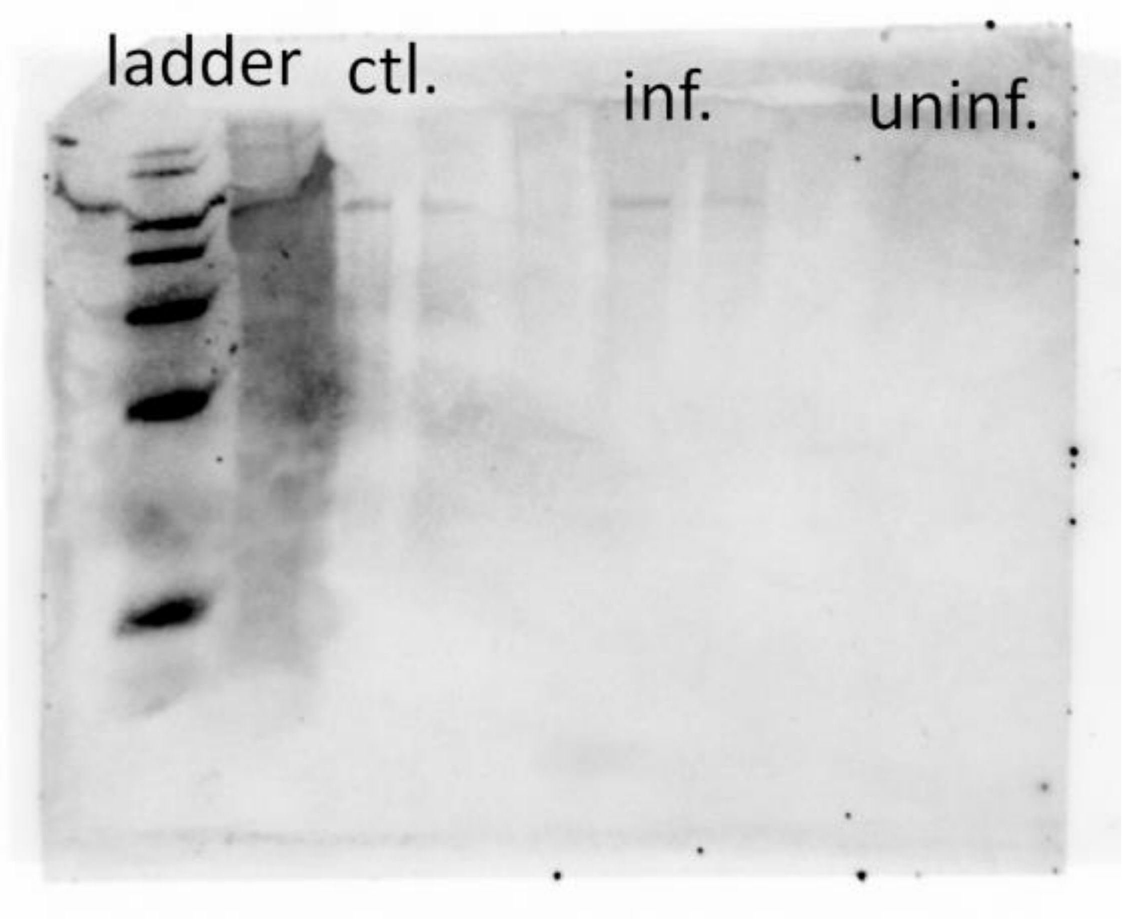

Fig. 1j

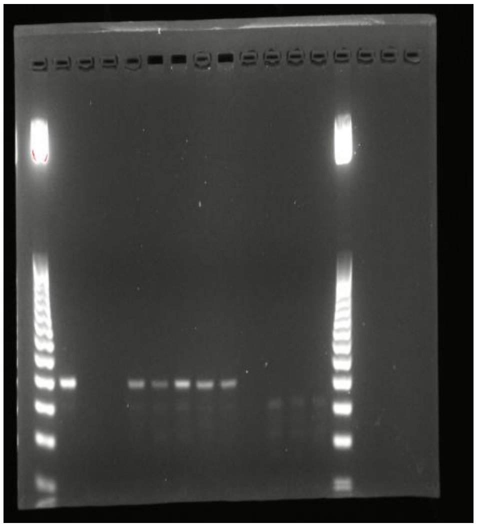

Supplement: Supplementary file 4 [file 41467_2026_77288_MOESM4_ESM.zip › Source Data for images.pdf]
